# A β-cap on the FliPQR protein-export channel acts as the cap for initial flagellar rod assembly

**DOI:** 10.1073/pnas.2507221122

**Published:** 2025-08-20

**Authors:** Miki Kinoshita, Tomoko Miyata, Fumiaki Makino, Katsumi Imada, Keiichi Namba, Tohru Minamino

**Affiliations:** ^a^Graduate School of Frontier Biosciences, Osaka University, Suita, Osaka 565-0871, Japan; ^b^JEOL YOKOGUSHI Research Alliance Laboratories, Osaka University, Suita, Osaka 565-0871, Japan; ^c^JEOL Ltd., Akishima, Tokyo 196-8558, Japan; ^d^Department of Macromolecular Science, Graduate School of Science, Osaka University, Toyonaka, Osaka 560-0043, Japan

**Keywords:** flagellum, cryoelectron microscopy, flagellar rod, type III secretion system

## Abstract

The FliPQR complex constitutes a channel for export of the flagellar proteins involved in axial structure assembly. It also serves as a template for the assembly of the rod structure, which consists of FliE, FlgB, FlgC, FlgF, and FlgG. FliP, FliQ, and FliR assemble into a right-handed helical structure within the central pore of the flagellar basal body MS-ring, and the complex has two gates on the cytoplasmic and periplasmic sides. The periplasmic gate, formed by the N-terminal α-helices of FliP and FliR, remains closed until six FliE subunits assemble onto FliP and FliR to form the first layer of the rod, but it has remained unclear how each FliE subunit opens the gate and assembles in the absence of the rod cap required for efficient assembly of other rod proteins. Here, we present a cryoelectron microscopy structure of the FliPQR complex in closed form at 3.0 Å resolution. A β-cap, formed by the N-terminal β-strands of FliP and FliR, is located at the top of the FliPQR complex and tightly seals the closed gate. The β-cap has a narrow pore that efficiently and accurately leads the first FliE subunit to its assembly site. Interactions of FliE with FliP and FliR induce a conformational change in FliP and FliR, with their N-terminal α-helices move up and outward to open the gate. Consequently, each of the N-terminal β-strands of FliP and FliR detaches from the β-cap one after another, thereby creating a docking site for the next FliE subunit to efficiently assemble.

## Significance

The FliPQR complex forms a channel for export of flagellar structural subunits. It also acts as a template for FliE assembly within the Membrane-supramembrane-ring. However, despite previous studies of the FliPQR complex, it remains elusive how FliE precisely assembles on the top of FliP and FliR. Using cryoelectron microscopy and mutational analyses, we present evidence suggesting that a β-cap, situated at the tip of the FliPQR complex, seals the periplasmic gate and facilitates the successive binding of FliE to FliP and FliR to form the first layer of the rod at the tip of the FliPQR complex. The periplasmic gate, initially closed like a floral bud, opens like a blooming flower in this dynamic process to initiate rod assembly.

*Salmonella enterica* serovar Typhimurium (hereafter *Salmonella*) employs the flagellar type III secretion system (hereafter referred to as fT3SS) to construct the axial structure of the flagellum, which is responsible for rapid swimming motility in liquid environments (1). The fT3SS consists of a transmembrane export gate complex made up of FlhA, FlhB, FliP, FliQ, and FliR and a cytoplasmic ATPase ring complex consisting of FliH, FliI, and FliJ (2, 3) (*SI Appendix*, Fig. S1). The export gate complex is located within the central pore of the basal body MS-ring and is powered by the transmembrane electrochemical gradient of protons (4, 5). The cytoplasmic ATPase ring complex associates with the C-ring (6) and acts as an activator that enables the export gate complex to become a proton-driven protein transporter (7, 8).

Five FliP subunits and one FliR subunit assemble into a right-handed helical structure with a narrow central pore. This pore serves as a channel for export of proteins that constitute several distinct parts of the flagellum (9–11). Four FliQ subunits peripherally associate on the outside of the FliP_5_–FliR_1_ complex (11–13). One FlhB subunit associates with the FliP_5_–FliQ_4_–FliR_1_ complex (hereafter FliPQR) to regulate the opening and closing of the cytoplasmic gate of the export channel (14, 15). FlhA assembles to form a homononameric ring with its transmembrane part possibly surrounding the FliPQR–FlhB complex (16, 17) and functions as a proton-driven export engine (18–21). The C-terminal cytoplasmic domains of FlhA and FlhB project into the cytoplasmic cavity of the C-ring. They form a docking platform for the cytoplasmic ATPase complex, the flagellar export chaperones, and the structural subunits of the flagellum (22–26). This docking platform ensures the proper order of flagellar protein export during the flagellar assembly process (27–31).

The rod is the drive shaft of the flagellar motor, transmitting the motor torque to the hook and filament in the cell exterior to produce thrust for cell swimming. It is a helical tubular structure formed by five different proteins, FliE, FlgB, FlgC, FlgF, and FlgG, with approximately 5.5 subunits per turn of the helix. It is composed of six FliE subunits, five FlgB subunits, six FlgC subunits, five FlgF subunits, and twenty-four FlgG subunits (12, 13). FliE is exported into the periplasm via the fT3SS (32) and forms the first helical layer of the rod on top of the FliP and FliR subunits, followed by the assembly of FlgB, FlgC, FlgF, and FlgG in this order (12, 13). Thus, the FliPQR complex also acts as a template for rod assembly.

FliE has unique features that distinguish it from the other four rod proteins. The *fliE* gene is adjacent to the *fliF* gene that encodes the MS-ring and is located at a different locus from other rod genes that forms the *flgBCDEFGHIJ* operon (1). Extensive interactions of FliE with FliP and FliR allow other flagellar proteins to efficiently diffuse through the central channel of the growing flagellar structure and assemble at the distal end (33). FliE has no common structural motifs found in other rod proteins (12, 13, 34). These properties suggest that FliE has a somewhat specialized function. Indeed, it has two distinct functional roles in the flagellar assembly process: first as a structural adapter that firmly anchors the rod to the MS-ring and the FliPQR complex and second as an export-channel activator of the fT3SS to fully open the export channel (35–37). FliE is composed of three α-helices: α1, α2, and α3. The N-terminal α1 helix of FliE binds to the inner wall of the MS-ring, whereas helices α2 and α3 form domain D0, which is the inner core domain of the flagellar axial structure. This D0 domain interacts with FliP, FliR, FlgB, and FlgC within the MS-ring (12, 13). Thus, FliE appears to couple the completion of export channel formation with the initiation of rod assembly. However, the mechanism by which it carries out this function remains to be elucidated. Furthermore, FliE can self-assemble directly on the top of FliP and FliR in the absence of the rod cap. This phenomenon stands in contrast to the other rod proteins, which require a cap complex for efficient incorporation into the rod structure at the growing end (38). However, the precise mechanism by which FliE efficiently and precisely assembles at the tips of FliP and FliR remains to be elucidated.

The FliPQR complex has two gates on the cytoplasmic and periplasmic sides. In the structure of the purified FliPQR complex (PDB ID: 6R69), the periplasmic gate of the export channel is closed by intermolecular interactions between the N-terminal α-helices of five FliP subunits and one FliR subunit. In the native basal body (PDB ID: 8WKK), however, the periplasmic gate is held open because of direct binding of six FliE subunits to these α-helices. Unfortunately, the N-terminal region of FliP has not been visualized in either the purified FliPQR complex or the native basal body (39, 40). As a result, the mechanism by which the closed periplasmic gate of the export channel opens upon FliE assembly has remained obscure.

To address this issue, we conducted cryoelectron microscopy (cryoEM) structural analysis of the purified *Salmonella* FliPQR complex reconstituted in a peptidisc, which is a sheet of short amphipathic bihelical peptides, and obtained the structure of the completely closed form at 3.0 Å resolution. We also conducted mutational analyses. We show that the periplasmic gate of the export channel is entirely sealed by the β-cap formed by the N-terminal β-strands of FliP and FliR. We also demonstrate that the interaction of FliE with FliP induces a conformational change in the MTSF motif of FliP (residues 61 to 64) that results in the outward movement of its N-terminal α-helix. This process sequentially detaches the β-strands from the β-cap to create the next FliE assembly site. Deletion analyses of residues 155 to 166 of FliP, which form a structural motif called p-loop on the surface of the FliPQR complex facing the inner wall of the MS-ring, demonstrated that the interaction of the p-loop with the MS-ring inner wall stabilizes the open conformation of its N-terminal α-helix. Based on these observations, we propose that the β-cap not only seals the periplasmic gate to limit uncontrolled protein secretion into the periplasm but also functions as a scaffold upon which the newly transported FliE subunits can efficiently assemble.

## Results

### CryoEM Structural Analysis of the FliPQR Complex Reconstituted in a Peptidisc.

**Fig. 1.**
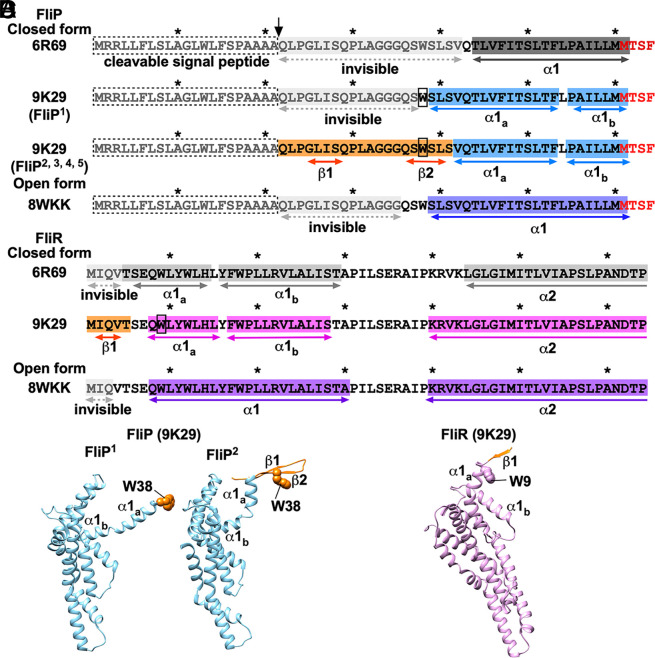
Amino acid sequences and atomic models of the N-terminal regions of FliP and FliR in the completely closed form of the FliPQR complex. (*A* and *B*) Amino acid sequences of the N-terminal regions of (*A*) FliP and (*B*) FliR. The N-terminal regions of FliP and FliR are in the closed conformation in the 6R69 and 9K29 structures, but in the open conformation in the 8WKK structure. The first 21 amino acid residues of FliP serve as the N-terminal signal peptide that is cleaved during membrane insertion. The arrow indicates that the cleavage occurs between Ala-21 and Gln-22. Residues 22 to 42 in the 6R69 structure and residues 22 to 35 in the 8WKK structure are invisible. However, these residues are visible in the 9K29 structure. The well-conserved MSTF motif is highlighted in red. The first 5 and 3 residues of FliR are not visible in the 6R69 and 8WKK structures, respectively, but are visible in the 9K29 structure. These visible regions, highlighted in orange, form the β-cap, which is stabilized by six tryptophan residues (Trp-38 of FliP and Trp-9 of FliR) indicated by open boxes. (*C*) Cα ribbon diagrams of the atomic models of two FliP subunits, the first (FliP^1^) and second (FliP^2^), obtained in this study (PDB ID: 9K29). (*D*) Cα ribbon diagram of the atomic model of FliR obtained in this study (PDB ID: 9K29).

FliP has a cleavable signal peptide (residues 1 to 21) at its N terminus. The signal peptide is cleaved during membrane insertion (41). Residues of 22 to 42 of mature FliP and residues 1 to 5 of FliR are not visible in the FliPQR complex solubilized with lauryl maltose neopentyl glycol (LMNG) (PDB ID: 6R69) (39) (Fig. 1 *A* and *B*). Because detergents often negatively affect protein structure and function, we decided to reconstitute the FliPQR complex into a peptidisc formed by short amphipathic bihelical peptides (42, 43). To efficiently and rapidly purify the FliPQR complex, a 10-residue His-tag was added to the C-terminus of FliR. The His-tag had no detectable effect on FliR function (*SI Appendix*, Fig. S2*A*). FliP, FliQ, and FliR–His were expressed from a pTrc99-based plasmid in a *Salmonella* strain SJW1368, in which the flagellar *flhDC* master operon required for the expression of all flagellar genes is deleted. Crude membranes were isolated by ultracentrifugation and solubilized by 1% (w/v) LMNG. The His-tagged FliPQR complex was purified by nickel affinity chromatography, followed by size exclusion chromatography. Then, we replaced LMNG by short amphipathic bihelical peptides (*SI Appendix*, Fig. S2*B*). Multiple copies of the peptide wrap around the transmembrane portion of the FliPQR complex to shield the hydrophobic surface, thereby making the complex very soluble in detergent-free solutions. This preparation yielded homogeneously dispersed particles readily visualized by negative-stain electron microscopy (negative-stain EM) and cryoEM (*SI Appendix*, Figs. S2*C* and S3*A*).

A total of 1,505,398 particles were extracted from 13,230 cryoEM micrographs and analyzed by single-particle image analysis (*SI Appendix*, Fig. S3*B*). After iterative 3D refinement with C1 symmetry, the 3D image of the FliPQR complex was reconstructed at 3.0 Å resolution from 109,333 particles (EMDB ID: EMD-61993) (*SI Appendix*, Fig. S3*C* and Table S1). The resolutions of the previous structures of FliPQR not incorporated into the flagellar basal body, 6S3R, 6S3S, 6S3L, 6F2D, and 6R69 were 3.5 Å, 4.1 Å, 3.2 Å, 4.2 Å, and 3.65 Å, respectively (11, 14, 39). Our present map represents a significant improvement in resolution and quality over these previous structures and has allowed us to build a more accurate atomic model of the FliPQR complex (PDB ID: 9K29).

**Fig. 2.**
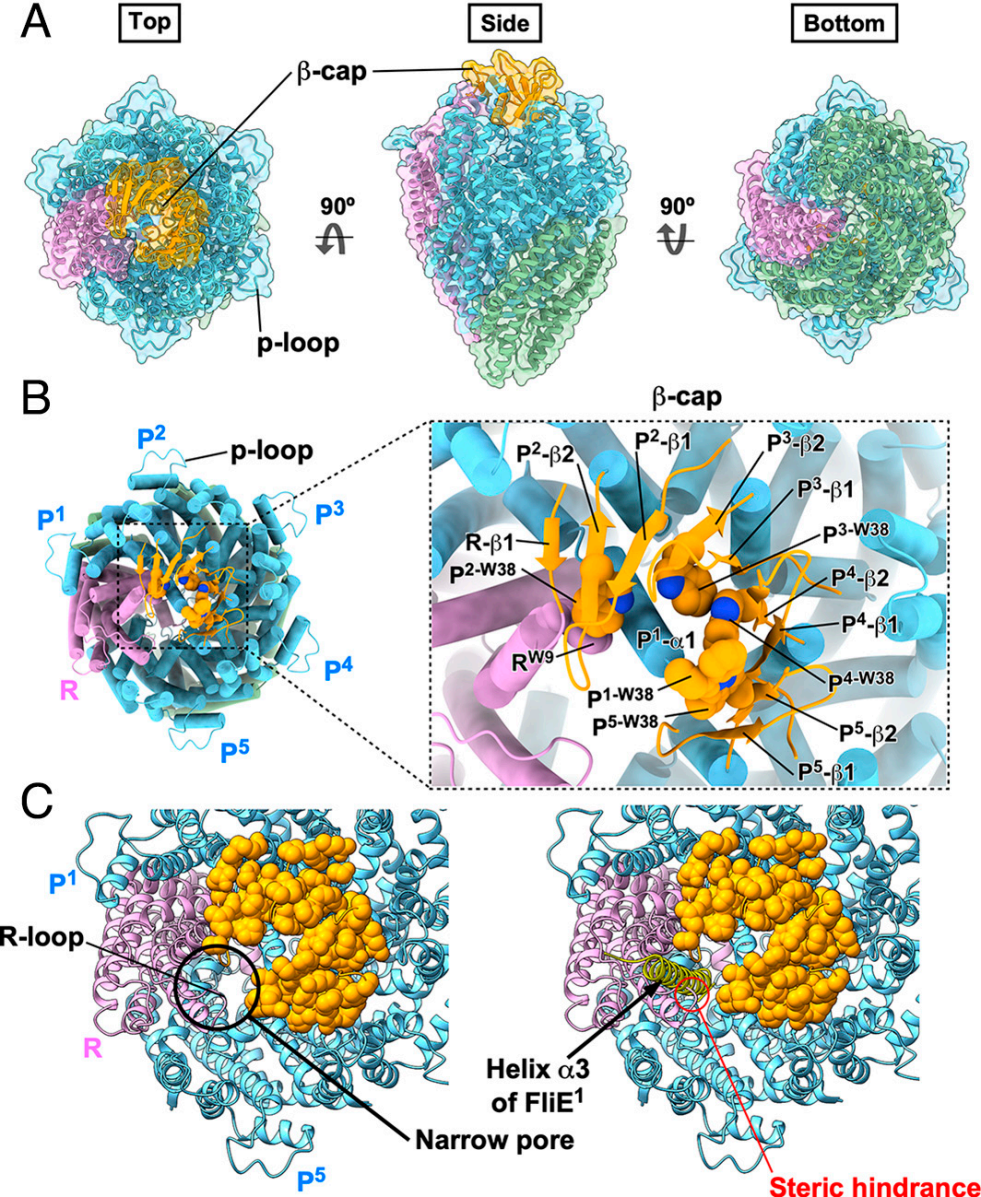
The cryoEM structure of the FliPQR complex reconstituted in the peptidisc. (*A*) Cα ribbon diagram of the atomic model of the FliPQR complex with a stoichiometry of 5 FliP, 4 FliQ, and 1 FliR subunits (PDB ID: 9K29). FliP, FliQ, and FliR are colored in sky blue, dark sea green, and plum, respectively. The extreme N-terminal regions of FliP and FliR, which are colored in orange, form the β-cap. The p-loop, consisting of residues 155 to 166, is located in the outermost part of the FliPQR complex. (*B*) The β-cap formed by the N-terminal β strands of FliP and FliR. The FliP subunit located at the top of the right-handed helical structure of the FliPQR complex is designated as the first FliP (FliP^1^) subunit. The remaining subunits, arranged along the helical staircase, are subsequently referred to as the second (FliP^2^), third (FliP^3^), fourth (FliP^4^), and fifth (FliP^5^) subunits, respectively. Four FliP subunits, FliP^2^, FliP^3^, FliP^4^, and FliP^5^, but not FliP^1^, contain two β-strands (β1, β2) forming a β-hairpin at their N-terminal regions. FliR possesses a β-strand (β1) in its extreme N-terminal region. The four β-hairpins and β1 of FliR form the β-cap, which is stabilized by hydrophobic interactions of six tryptophan residues (Trp-38 of FliP and Trp-9 of FliR). The extreme N-terminal region of α1 of FliP^1^ project into the cavity of the β-cap. (*C*) The narrow pore within the β-cap. The N-terminal region of FliP^1^ is not involved in the β-hairpin formation, which leaves a narrow pore between the FliR and FliP^5^ subunits (*Left*). Comparison of the 9K29 structure with the 8WKK structure by superposition reveals that the pore is sufficiently wide to accommodate helix α3 of the first FliE subunit (FliE^1^, yellow), which is inserted between FliR and FliP^5^ in the 8WKK structure (*Right*). Side-chain atoms of FliP and FliR involved in the formation of the β-cap are indicated by orange spheres. The R-loop formed by residues 55 to 68 of FliR extends into the pore, where it exhibits an apparent steric clash with the α-helix as marked by a red circle.

In agreement with previous reports, the FliPQR complex adopts a right-handed helical structure composed of 5 FliP subunits, 4 FliQ subunits, and 1 FliR subunit (Fig. 2*A*). The five FliP subunits assemble into a helical array, with FliP^1^ at the top and FliP^2^–FliP^5^ following a descending spiral. The densities corresponding to residues 22 to 41 of four mature FliP subunits, FliP^2^, FliP^3^, FliP^4^, and FliP^5^, and residues 1 to 5 of FliR are clearly visible, enabling us to build the atomic model of these N-terminal regions (Fig. 1 *C* and *D*). The density corresponding to residues 22 to 37 of FliP^1^ is invisible. The N-terminal region of the four FliP subunits whose N termini are resolved forms a β-hairpin with β-strands β1 and β2. The N-terminal region of FliR contains a single β-strand (β1). These β-strands form a β-sheet at the tip of the FliPQR complex, which we named the β-cap, (Fig. 2*B* and *SI Appendix*, Fig. S4). A total of six tryptophan residues, five Trp-38 of FliP and Trp-9 of FliR, stabilize the β-cap structure via hydrophobic contacts with each other. Furthermore, the N-terminal α-helix (α1) of FliP^1^ projects toward the export channel, and its N-terminal region, including Trp-38, inserts into the cavity of the β-cap, thereby maintaining the closed state of the periplasmic gate. Consequently, our cryoEM structure of the FliPQR complex adopts a bud-like shape (Fig. 2 *A*, *Middle*).

**Fig. 3.**
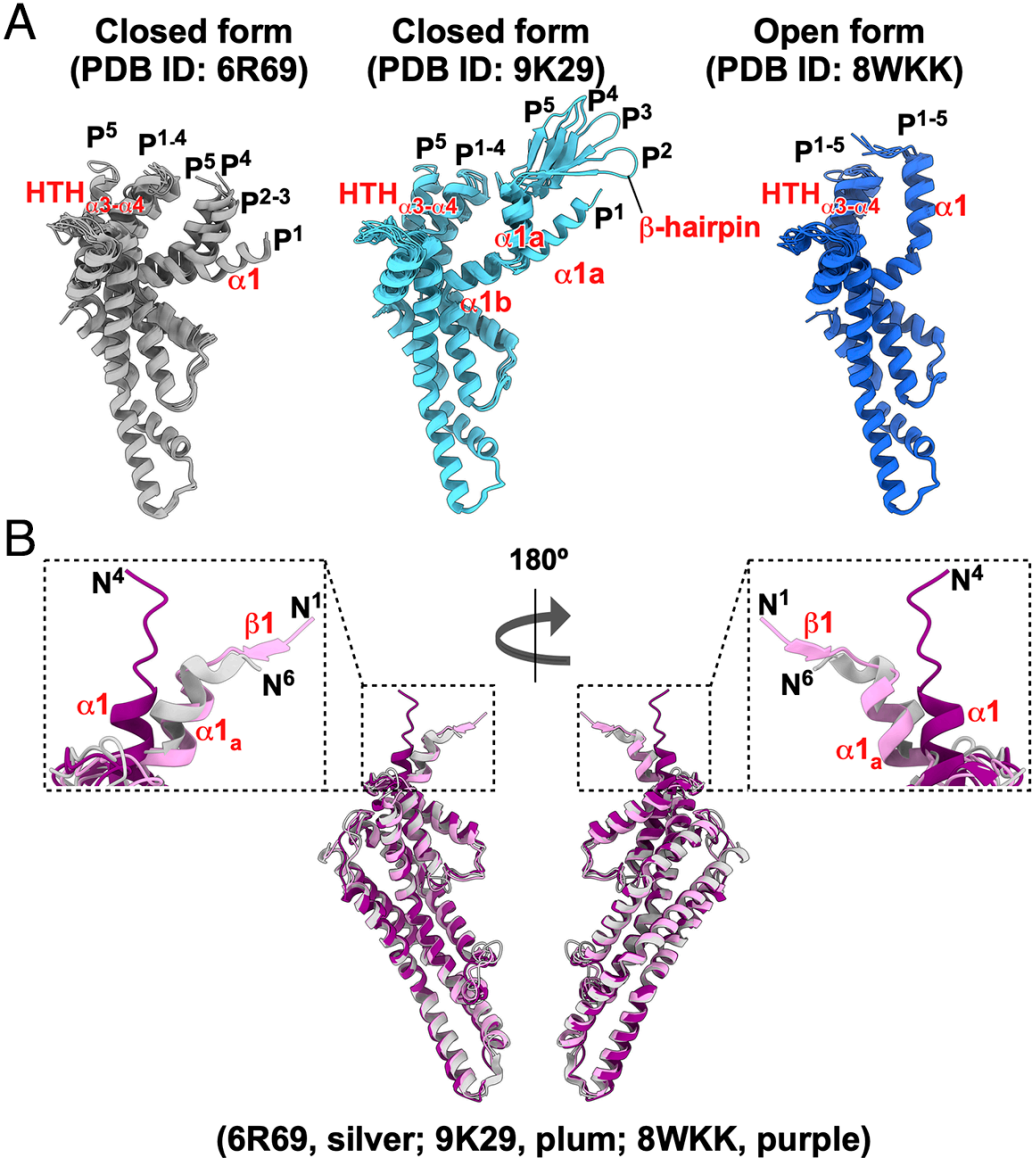
Structural comparisons of FliP and FliR subunits in the FliPQR complex of the present study (PDB ID: 9K29) with those of previous studies. (*A*) Structural comparisons of the five FliP subunits in the three different FliPQR structures. The RMS deviations (RMSDs) for these superpositions are provided in *SI Appendix*, Table S2. The conformations of the five FliP subunits are slightly different from each other in the 6R69 (dark gray) and 9K29 (sky blue) structures, whereas the five FliP subunits are nearly identical to each other in the 8WKK structure (royal blue). In the 9K29 structure, the N-terminal β-hairpin structure is visible in FliP^2^, FliP^3^, FliP^4^, and FliP^5^, but not in FliP^1^. The orientation of α1 is different among the five FliP molecules in the 6R69 and 9K29 structures. In the FliP^5^ subunit, the helix–turn–helix structure (HTH_α3-α4_) formed by α3 and α4 and a loop connecting these two helices is shifted outward relative to the other four FliP subunits. In the 8WKK structure, the orientation of α1 and the position of HTH_α3-α4_ are nearly identical among the five FliP molecules. (*B*) Structural comparison of the FliR subunits in the three different FliPQR structures. The FliR subunits from the 6R69 and 8WKK structures were individually superimposed onto the equivalent coordinate in the 9K29 structure. The RMSD values for these superpositions are listed in *SI Appendix*, Table S3. The overall structure of FliR is almost identical among the 6R69 (silver), 9K29 (plum), and 8WKK (purple) structures. However, in the 8WKK structure, the N-terminal α1 helix adopts a different orientation compared to those in the 6R69 and 9K29 structures. The N-terminal β-strand (β1) is visible only in the 9K29 structure.

Helix α1 of FliP^1^ is longer by three residues (S39-L40-S41) than helix α1 of the remaining four FliP subunits (Fig. 1*A*), indicating that the S39-L40-S41 region is convertible between α-helix and β-strand. Helix α1 is kinked at Leu-54 and is divided into two distinct parts, designated as α1_a_ and α1_b_ (Fig. 1*C*). Except for FliP^1^, α1_a_ rises upward and moves outward. In addition, the β-hairpins of the FliP^2^, FliP^3^, FliP^4^, and FliP^5^ subunits also rise upward (Fig. 3 *A*, *Middle*), enabling them to form the β-cap together with β1 of FliR (Fig. 2*B*).

The β-cap has a narrow pore between FliP^5^ and FliR that is wide enough to allow the passage of a single α-helix (Fig. 2 *C*, *Left*). The FliE-Bla fusion protein is secreted via the fT3SS into the periplasm to a significant degree even in the absence of FliE, suggesting that FliE can pass through the pore (37). When our 9K29 structure is superimposed onto the corresponding part of a basal body structure, 8WKK, it is very clear that the pore can properly and precisely accommodate helix α3 of the first FliE subunit (FliE^1^) at the first FliE assembly site (Fig. 2 *C*, *Right*). Therefore, we propose that the β-cap not only keeps the periplasmic gate closed until FliE subunits assemble onto FliP and FliR but also serves as a scaffold for precise and efficient initiation of FliE assembly.

An apparent steric clash is observed between the P64-L65-F66 region within the R-loop of FliR (residues 55 to 68) and helix α3 of FliE^1^ within the pore of the β-cap (Fig. 2*C* and *SI Appendix*, Fig. S5). The secretion rate of the FliE-Bla fusion protein is increased by approximately fourfold in the presence of FliE (37). Furthermore, FlgD and FlgE cannot be efficiently transported via the fT3SS into the periplasm in the absence of FliE (33, 35, 36). Because such a steric clash is not observed in the 8WKK structure, we propose that the FliR loop may initially block premature secretion of the hook-capping protein FlgD and the hook protein FlgE until FliE assembles onto the FliPQR complex and that a local rearrangement of this loop allows proper accommodation of FliE during the assembly process.

### Structural Comparison of the Open and Closed Forms of the FliPQR Complex.

The α1 helices of five FliP subunits and one FliR subunit not only serve as a periplasmic gate but also provide assembly sites for the six FliE subunits (39, 40). The periplasmic gate of the export channel is closed in the 6R69 and 9K29 structures, but it is open in the 8WKK structure (*SI Appendix*, Figs. S4 and S6). To investigate how the β-cap disassembles when the periplasmic gate adopts the open conformation, we superimposed the FliP, FliQ, and FliR subunits of our structure (PDB ID: 9K29) onto the corresponding ones in the 6R69 and 8WKK structures. The overall structures are nearly identical to one another except for the conformation and orientation of the N-terminal regions of FliP and FliR (Fig. 3 and *SI Appendix*, Figs. S7 and S8 and Tables S2−S4).

Helix α1 of all five FliP subunits in the 6R69 structure is five residues shorter than that of FliP^1^ in the 9K29 structure and two residues shorter than those observed in the remaining four FliP subunits (Fig. 1*A*). Furthermore, the first four residues of FliR are invisible in the 6R69 structure (Fig. 1*B*). These observations suggest that the β-cap stabilizes the intermolecular FliP–FliP and FliP–FliR interactions, which in turn ensures that α1 is maintained in its closed conformation. In the 8WKK structure, the length of α1 observed for all five FliP subunits is the same as that of FliP^1^ in the 9K29 structure. In the native basal body, the extreme N-terminal region of α1 is stabilized by the interaction with FliE.

The orientation of α1 is different among the five FliP molecules in the closed structures of 6R69 and 9K29. In the FliP^5^ subunit, the helix–turn–helix structure (HTH_α3-α4_), which is formed by α3 and α4, and a loop connecting these two helices, is shifted outward relative to the other four FliP subunits (Fig. 3 *A*, *Left* and *Middle*). Conversely, in the open structure of 8WKK, the orientation of α1 and the position of HTH_α3-α4_ are nearly identical among the five FliP molecules (Fig. 3 *A*, *Right* and *SI Appendix*, Table S2). When each of the five FliP subunits in the 6R69 and 8WKK structures is superimposed on the corresponding FliP subunit in the 9K29 structure, α1 rises upward and HTH_α3-α4_ moves outward in the 8WKK structure (*SI Appendix*, Fig. S7 and Table S3).

The I2-Q3-V4 sequence of FliR forms strand β1 in the 9K29 structure, whereas the M1-I2-Q3 sequence is invisible in the 8WKK structure (Fig. 1*B*). This indicates that β1 is stable only in the β-cap. Helix α1 of FliR is kinked at Tyr-16 in the 9K29 structure, thereby dividing it into two distinct parts, named α1_a_ and α1_b_ (Fig. 1*D*). This kink allows β1 to form an antiparallel β-sheet with β2 of FliP^2^ in the β-cap. Additionally, it enables α1_a_, including Trp-9, to orient toward the export channel to stabilize the β-cap (Fig. 2*B*). In the 8WKK structure, α1 of FliR moves outward as this kink disappears (Fig. 3*B*).

### Mutational Analysis of Trp-38 of FliP and Trp-9 of FliR.

Trp-38 of FliP and Trp-9 of FliR stabilize the β-cap (Fig. 2*B*). Conservation analysis using ConSurf indicated that Trp-38 of FliP is poorly conserved across 150 bacterial species, whereas Trp-9 of FliR is moderately conserved (*SI Appendix*, Fig. S9*A*). Whether these Trp residues are critical for proper periplasmic channel formation remains an open question. To address this question, we constructed the *fliP(W38A)*, *fliP(W38G)*, *fliR(W9A)*, and *fliR(W9G)* mutants and analyzed the secretion of FlgD, a representative export substrate of the fT3SS, by immunoblotting with polyclonal anti-FlgD antibody. The W38A and W38G substitutions in FliP markedly reduced the secretion level of FlgD, without affecting the expression level of FliP (*SI Appendix*, Fig. S9 *B*, *Left*). In contrast, W9A and W9G substitutions in FliR caused only a slight reduction in FlgD secretion (*Right*). However, it was not possible to tell whether this reduction was caused by these two mutations because immunoblotting failed to detect cellular FliR and therefore the levels of FliR could not be compared between the wild-type and two mutants. These results suggest that Trp-38 of FliP is critical for proper export channel formation, whereas the role of Trp-9 of FliR remains unclear.

### Mutational Analysis of the MTSF Motif of FliP.

**Fig. 4.**
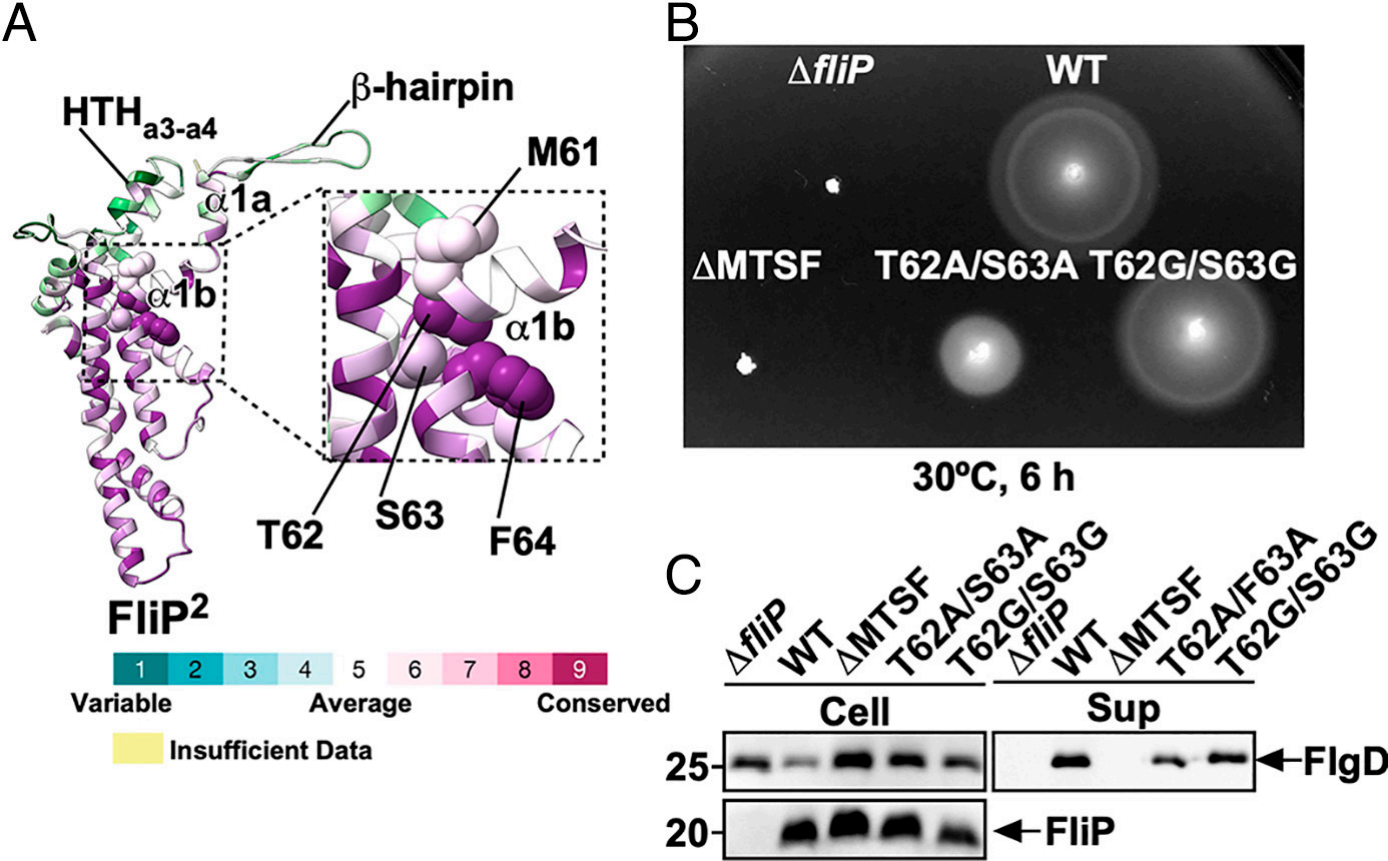
Mutational analysis of the conserved MTSF motif in FliP. (*A*) Evolutionarily conserved residues of FliP. Conservation scores were calculated using the ConSurf web server. Residues are colored according to sequence conservation among 150 bacterial species. (*B*) Motility assay of a *Salmonella fliP* null mutant carrying pTrc99AFF4 (∆*fliP*), pKY69 (WT), pMKM69(∆MTSF) (∆MTSF), pMKM69(T62A/S63A) (T62A/S63A), or pMKM69(T62G/S63G) (T62G/S63G) in soft agar. The plates were incubated at 30 °C for 6 h. At least seven independent assays were performed. (*C*) Secretion assay. Whole-cell proteins (Cell) and culture supernatants (Sup) were prepared from the above strains. A 5 μL aliquot of each protein sample, normalized to OD_600_, was subjected to SDS-PAGE, followed by immunoblotting with polyclonal anti-FlgD (first row) and anti-FliP (second row) antibodies. Molecular mass markers (kDa) are shown on the *Left*. At least three independent assays were carried out.

Until FliE binds to FliP and FliR, the periplasmic gate of the export channel resembles a closed flower bud because of the β-cap at the tip of the FliPQR complex. When FliE interacts with FliP and FliR, the periplasmic gate opens completely, resembling a flower in full bloom. Comparison of the open and closed conformations of FliP reveals that, although the conformational changes between the open and closed structures differ slightly from subunit to subunit, a consistent conformational change in the region of residues 59 to 64 is observed in all five subunits. Met-61, Thr-62, Ser-63, and Phe-64 (MTSF) are conserved among FliP homologues (Fig. 4*A* and *SI Appendix*, Fig. S10*A*). They form a hinge that allows helix α1 of FliP to move upward when the FliPQR complex assumes the open conformation (Movie S1). To investigate whether a conformational rearrangement of the MTSF motif is necessary to open the periplasmic gate completely, we generated a *fliP* mutant lacking the MTSF motif (∆MTSF) and analyzed its motility in soft agar (Fig. 4*B*). Immunoblotting using polyclonal anti-FliP antibody revealed that the deletion did not affect the cellular level of FliP (Fig. 4*C*). However, unlike wild-type FliP, the ectopic production of FliP(∆MTSF) failed to restore motility in the ∆*fliP* mutant (Fig. 4*B*). Furthermore, immunoblotting with polyclonal anti-FlgD antibody revealed that the MTSF deletion inhibits FlgD secretion (Fig. 4*C*). Thus, the MTSF motif of FliP is necessary for the initiation of flagellar protein export.

To test whether the conformational flexibility of the MTSF motif is a prerequisite for the movement of α1, we generated two *fliP* mutants, *fliP(T62A/S63A)* and *fliP(T62G/S63G)*. The T62A/S63A double substitution caused a decrease in both motility and FlgD secretion. However, the T62G/S63G double substitution had no impact on either motility or FlgD secretion (Fig. 4 *B* and *C*), indicating that the specific side-chain interactions are not critical for the feature enabling the necessary conformational changes of the α1 helix. Because these two double mutations did not affect the cellular level of FliP (Fig. 4*C*), we suggest that the important function of the MTSF motif is to provide the conformational flexibility that allows α1 of FliP to move in a direction that completely opens the periplasmic gate.

To confirm this, we also constructed the *fliP(M61G/T62G/S63G/F64G)* (referred to as GGGG) and *fliP(M61G/T62S/S63G/F64S)* (referred to as GSGS) mutants. The GGGG and GSGS substitutions markedly reduced the cellular expression level of FliP, resulting in impaired motility and flagellar protein export (*SI Appendix*, Fig. S10 *B* and *C*). These results suggest that the MTSF motif also plays a critical role in stabilizing the FliP structure.

### Mutational Analysis of the Conserved Leu-92 Residue of FliP.

**Fig. 5.**
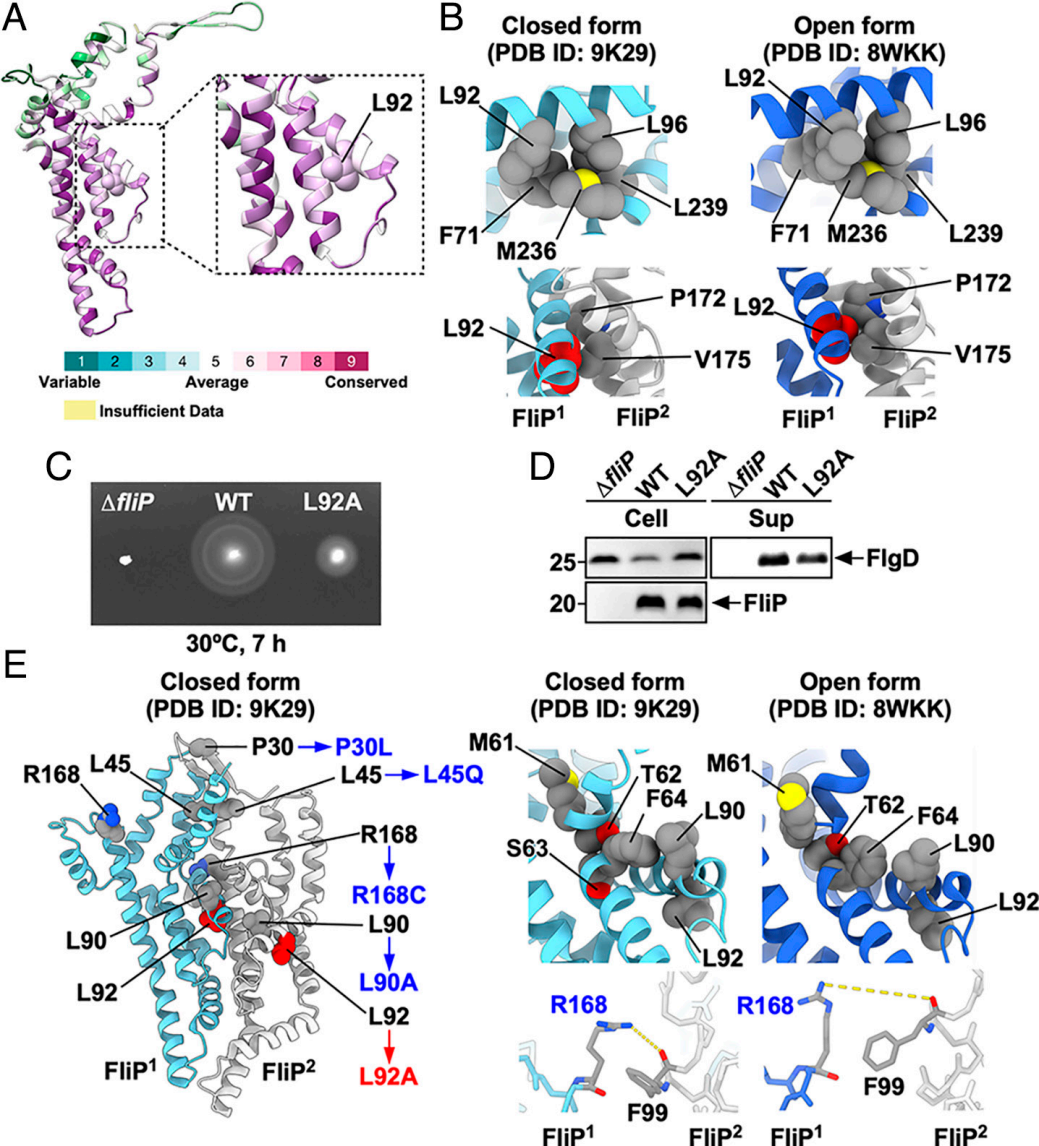
Role of the conserved Leu-92 residue of FliP in flagellar protein export. (*A*) Evolutionarily conserved residues of FliP. Conservation scores were calculated using the ConSurf web server (https://consurf.tau.ac.il/consurf_index.php). Residues are colored according to sequence conservation among 150 bacterial species. (*B*) Location of Leu-92 in the hydrophobic side-chain interaction networks in the closed (*Left*) and open (*Right*) conformations of the FliPQR complex. The interaction between FliE and FliP causes a conformational transition from the closed to the open state of FliP. Leu-92 forms an intramolecular hydrophobic interaction network with Phe-71, Leu-96, Met-236, and Leu-239. The FliE–FliP interaction induces remodeling of the hydrophobic interaction network formed by Phe-71, Leu-92, Leu-96, Met-236, and Leu-239 (*Upper*). Leu-92 of FliP^1^ also makes intermolecular hydrophobic contacts with both Pro-172 and Val-175 of FliP^2^ in the open form, but only with Val-175 in the closed form (*Lower*). (*C*) Motility assay of a *Salmonella fliP* null mutant carrying pTrc99AFF4 (indicated as ∆*fliP*), pKY69 (indicated as WT), or pMKM69(L92A) (indicated as L92A) in soft agar. The plates were incubated at 30 °C. At least seven independent assays were carried out. (*D*) Secretion assay. Whole-cell proteins (Cell) and culture supernatants (Sup) were prepared from the above strains. A 5 μL aliquot of each protein sample, normalized to OD_600_, was subjected to SDS-PAGE, followed by immunoblotting with polyclonal anti-FlgD (first row) and anti-FliP (second row) antibodies. Molecular mass markers (kDa) are shown on the *Left*. At least three independent experiments were performed. (*E*) Location of intragenic suppressor mutation sites identified in up-motile mutants isolated from the *fliP(L92A)* mutant. The L92A mutation site and its intragenic suppressor mutation sites are highlighted in red and blue, respectively. Pro-30 is located within the β-cap in the closed structure. Leu-45 forms a hydrophobic core with surrounding residues in helix α1, contributing to the closure of the periplasmic gate. Leu-90 engages in hydrophobic interaction with Phe-64 in the MSTF motif when FliP is in the closed form. Leu-45 and Leu-90 are also involved in the FliP–FliE interaction in the open structure (Also see *SI Appendix*, Fig. S6*B*). Arg-168 of FliP^1^ forms a hydrogen bond with the carbonyl oxygen of Phe-99 of FliP^2^ in the closed conformation but not in the open conformation.

The hydrophobic portions of the MTSF motif form a network of hydrophobic interactions with surrounding residues, thereby stabilizing a closed conformation of the MTSF motif (*SI Appendix*, Fig. S11). This observation raises the possibility that conformational changes in the MTSF motif are mediated through reorganization of these hydrophobic interactions. Given that Leu-92 of FliP is relatively well conserved among 150 bacterial species (Fig. 5*A*) and is involved in the remodeling of the hydrophobic interactions (Fig. 5*B*), we generated a *fliP(L92A)* mutant to investigate its functional role. The L92A substitution markedly reduced motility in soft agar (Fig. 5*C*). Consistently, this alanine substitution decreased the secretion level of FlgD by approximately twofold without affecting the cellular level of FliP (Fig. 5*D*). These results indicate that Leu-92 is critical for FliP function.

To determine whether the L92A substitution affects the assembly of the FliPQR complex, we purified the FliP(L92A)QR–His complex using Ni affinity chromatography, followed by size exclusion chromatography (*SI Appendix*, Fig. S12 *A* and *B*). FliP(L92A) and FliQ were copurified with FliR–His in a manner comparable to the wild-type FliPQR–His complex. Negative-stain EM revealed that they form a complex structure similar to that of the wild type (*SI Appendix*, Fig. S12*C*). These results indicate that the L92A mutation does not disrupt FliPQR complex formation, supporting the interpretation that the observed effects on motility and flagellar protein export results from alterations in export gate function rather than impaired complex formation.

Leu-92, situated at the interface of the FliP subunits, forms hydrophobic interactions with Val-175 of the neighboring FliP subunit in the closed form and with both Pro-172 and Val-175 of the neighboring FliP subunit in the open form (Fig. 5*B*). Thus, Leu-92 may enhance hydrophobic interactions between the FliP subunits in the open structure of the FliPQR complex. The L92A substitution reduces the stability of the open conformation of FliP, thereby increasing the likelihood that helix α1 will adopt a closed conformation. To confirm this, we calculated the buried surface area (BSA) of Leu-92 in both the closed and open conformations of the FliPQR complex (*SI Appendix*, Table S5). In the open conformation, the BSA of Leu-92 in FliP^2^ was 69.49 Å^2^ and decreased to 24.42 Å^2^ upon substitution with alanine (L92A). In contrast, in the closed conformation, the BSA of Leu-92 in FliP^2^ was 46.80 Å^2^, and the L92A mutation reduced it to 36.76 Å^2^. These results suggest that the destabilizing effect of the L92A mutation is more pronounced in the open conformation than in the closed state.

Differences in the hydrophobic interaction mode of Leu-92 between the open and closed states of the FliPQR complex can be attributed to two factors: the 0.5-turn shift of the α-helix containing Val-175 relative to Leu-92 and the different orientation of the side chain of Leu-92. Therefore, we introduced four sets of *fliP* mutations that cause the residue substitutions, L90A/G91A, L90A/L92A, G91A/L92A, and L90A/G91A/L92A. The motility of the *fliP(L90A/L92A)* and *fliP(L90A/G91A/L92A)* mutants was much better than that of the *fliP(L92A)* mutant although not as good as that of the wild type (*SI Appendix*, Fig. S13). Because the G91A substitution alone did not improve the motility of the *fliP(L92A)* mutant, we conclude that the L90A substitution allows FliP(L92A) to adopt a stable open conformation. In the closed structure of 9K29, Leu-90 establishes an intramolecular hydrophobic contact with Phe-64 in the MTSF motif (Fig. 5*E*). In contrast, Leu-90 interacts with Met-102 of FliE in the open structure (*SI Appendix*, Fig. S6*B*). Therefore, we suggest that the interaction between FliP and FliE induces a restructuring of the hydrophobic side-chain networks surrounding the MTSF motif, thereby opening the periplasmic gate of the export channel.

We also isolated intragenic suppressor mutants from the *fliP(L92A)* mutants. DNA sequencing revealed that the *fliP(P30L)*, *fliP(L45Q)*, and *fliP(R168C)* mutations restored the motility of the *fliP(L92A)* mutant (*SI Appendix*, Fig. S13). Pro-30 is located within the β-cap, which serves to stabilize the closed conformation of the periplasmic gate (Fig. 5*E*). Leu-45 is situated at the core of the hydrophobic region between the α1 helices of FliP subunits in the closed form of the FliPQR complex (*SI Appendix*, Fig. S6*A*). Arg-168 forms a hydrogen bond with the carbonyl group of Phe-99 of the adjacent FliP subunit in the closed form. However, this hydrogen bond is absent in the open form (Fig. 5*E*). This indicates that Arg-168 plays a role in stabilizing the closed conformation of FliP. Therefore, these three residues stabilize the closed conformation, and the P30L, L45Q, and R168C substitutions destabilize the closed conformation of the periplasmic gate even in the presence of the *fliP*(L92A) mutation.

### Interaction between FliF and FliP.

**Fig. 6.**
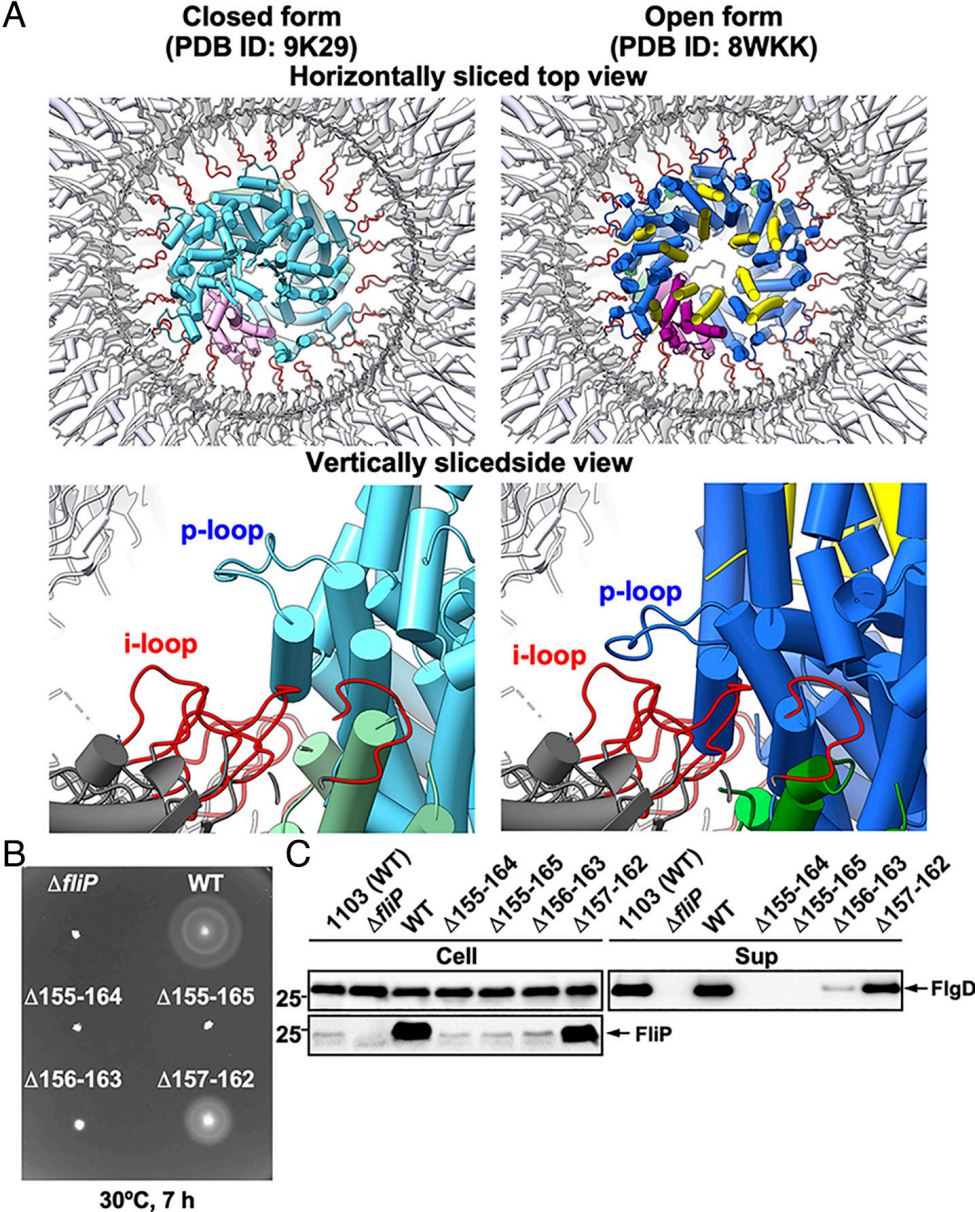
Effect of in-frame deletions of the p-loop of FliP on flagellar protein export. (*A*) Structural comparison of the p-loop conformations in the closed (PDB ID: 9K29) and open (PDB ID: 8WKK) forms of the FliPQR complex. The open and closed structures are superimposed on the equivalent coordinates of the 7NVG structure. FliP, FliQ, and FliR are colored in sky blue, dark sea green, and plum, respectively, in the 9K29 structure. FliE, FliP, FliQ, and FliR are colored in yellow, royal blue, green, and purple, respectively, in the 8WKK structure. The FliPQR complex is located within the central pore of the MS-ring. Each p-loop (residues 155 to 166) makes physical contact with the i-loop of the MS-ring protein FliF (red, residues 159 to 172) within the MS-ring after 6 FliE subunits assemble on FliP and FliR. (*B*) Motility assay of a *Salmonella fliP* null mutant carrying pTrc99AFF4 (indicated as ∆*fliP*), pKY69 (indicated as WT), pMKM69(∆155-164) (indicated as ∆155-164), pMKM69(∆155-165) (indicated as ∆155-165), pMKM69(∆156-163) (indicated as ∆156-163), or pMKM69(∆157-162) (indicated as ∆157-162) in soft agar. The plates were incubated at 30 °C for 7 h. At least seven independent assays were carried out. (*C*) Secretion assay. Whole-cell proteins (Cell) and culture supernatants (Sup) were prepared from the above transformants. A 5 μL aliquot of each protein sample, normalized to OD_600_, was subjected to SDS-PAGE, followed by immunoblotting with polyclonal anti-FlgD (first row) and anti-FliP (second row) antibodies. Molecular mass markers (kDa) are shown on the *Left*. At least three independent assays were performed.

The i-loop of FliF formed by its residues 159 to 172 is required for assembly of the export gate complex in the central pore of the MS-ring (44, 45). Residues 155 to 165 of FliP (p-loop) engage with the i-loop when the export gate complex is inserted into the central pore of the MS-ring with FliP adopting an open conformation (Fig. 6 *A*, *Right*). However, the p-loop may not interact with the i-loop in the same manner when FliP adopts a closed conformation (Fig. 6 *A*, *Left*). This raises the possibility that the interaction between the p-loop of FliP and the i-loop of FliF stabilizes the open conformation of the periplasmic gate.

To test this hypothesis, we constructed four *fliP* deletion mutants, ∆155 to 164, ∆155 to 165, ∆156 to 163, and ∆157 to 162. Deletion of residues 157 to 162 decreased both motility (Fig. 6*B*) and FlgD secretion slightly (Fig. 6*C*) but did not decrease the level of FliP. Thus, residues 157 to 162 are required for efficient assembly of the flagellar protein export gate in the pore of the MS-ring but not for stability of FliP.

The larger deletions had more severe effects (Fig. 6 *B* and *C*). The ∆156 to 163 mutant had markedly diminished motility and reduced FlgD secretion, and this was possibly due to a significant reduction in the cellular FliP level. Thus, deletion of the two residues immediately adjacent to residues 157 to 162 severely reduced the stability of FliP. The even larger deletions ∆155 to 164 and ∆155 to 165 resulted in a completely nonmotile phenotype as well as a more significant decrease in the cellular level of FliP. However, the FliP levels of these three deletion variants were still comparable to the level of FliP expressed from the chromosomal *fliP* gene. These results show that the intact p-loop is required for FliP to adopt the open conformation upon FliE assembly onto FliP and FliR. The decreased stability of FliP in the ∆155 to 164, ∆155 to 165, and ∆156 to 163 mutants additionally shows that some elements of the p-loop are also required for proper folding of FliP.

## Discussion

It has previously been shown that the FliPQR complex provides a channel for the export of proteins that assemble into the rod, the hook, and the filament and that it provides a structural template for rod assembly within the MS-ring (12, 13). The N-terminal α1 helices of FliP and FliR serve as a periplasmic gate of the export channel. The extensive hydrophobic interactions between these helices close the periplasmic gate until FliE assembles onto FliP and FliR to form the first layer of the proximal rod at the tip of the FliPQR complex (*SI Appendix*, Fig. S6). The assembly of FliE also opens the periplasmic gate to allow export of the proteins that compose the rod, the hook, and the filament (11–13, 37). Thus, FliE assembly is an important checkpoint for the sequential flagellar assembly process. Because FliE can efficiently self-assemble at the tips of FliP and FliR without the help of the rod cap that is required for efficient and robust rod formation (32, 33, 35, 38), the detailed mechanism for opening the periplasmic gate and initiating rod assembly was not fully understood.

We therefore performed high-resolution cryoEM image analysis of the FliPQR complex reconstituted in peptidisc. This allowed us to visualize the β-cap at the tip of the FliPQR complex (Fig. 2). The β-cap is stabilized by hydrophobic interactions between Trp-38 of five FliP subunits and Trp-9 of one FliR subunit. In addition, the extreme N terminus of α1 of one FliP protein (FliP^1^) projects into the cavity of the β-cap, providing a tight seal for the periplasmic gate (Fig. 2*B*).

To determine how the interactions of FliP and FliR with FliE disrupt the β-cap and allow the α1 helices of FliP and FliR to adopt the conformation they take in the open gate, we performed mutational analyses. The conserved Leu-92 residue stabilizes the open conformation through interactions with Pro-172 and Val-175 of its neighboring FliP subunit (Fig. 5*B*). The L92A substitution removes these hydrophobic contacts and causes the gate to remain closed much of the time (*SI Appendix*, Table S5), consequently impairing flagellar protein export and motility significantly (Fig. 5 *C* and *D*).

In the closed gate, Leu-90 of FliP makes an intramolecular hydrophobic contact with Phe-64 within the MTSF motif (Fig. 5*E*) but establishes a hydrophobic interaction with Met-102 of FliE in the open form (*SI Appendix*, Fig. S6*B*). Pro-30 is located within the β-hairpin and stabilizes the β-cap in the closed form (Fig. 5*E*). In the closed form, Leu-45 is in helix α1, where it forms a hydrophobic core with Phe-47 and Leu-51 that keeps the export channel closed (*SI Appendix*, Fig. S6*A*). In the open form, helix α1 including Leu-45 interacts with FliE (*SI Appendix*, Fig. S6*B*).

The conformational flexibility of the conserved MTSF motif of FliP is necessary for opening the periplasmic gate (Fig. 4 and *SI Appendix*, Fig. S10). It seems that the extensive hydrophobic interactions of FliP with FliE induce a remodeling of the hydrophobic side-chain networks surrounding the MTSF motif. These changes allow Leu-92 to interact with both Pro-172 and Val-175 of its neighboring FliP subunit to stabilize the conformation of the MTSF motif in the open gate (Fig. 5*B*). This promotes the release of the β-hairpin from the β-cap, and helix α1 of FliP adopts the conformation that it assumes in the open gate. Furthermore, the direct contact of each p-loop with the i-loop of FliF locks the open state of each FliP subunit within the MS-ring (Fig. 6). The second-site substitutions P30L, L45Q, L90A, and R168C destabilize the closed conformation conferred by FliP(L92A), thereby improving the motility of the *fliP*(L92A) mutant to a significant degree (*SI Appendix*, Fig. S13).

**Fig. 7.**
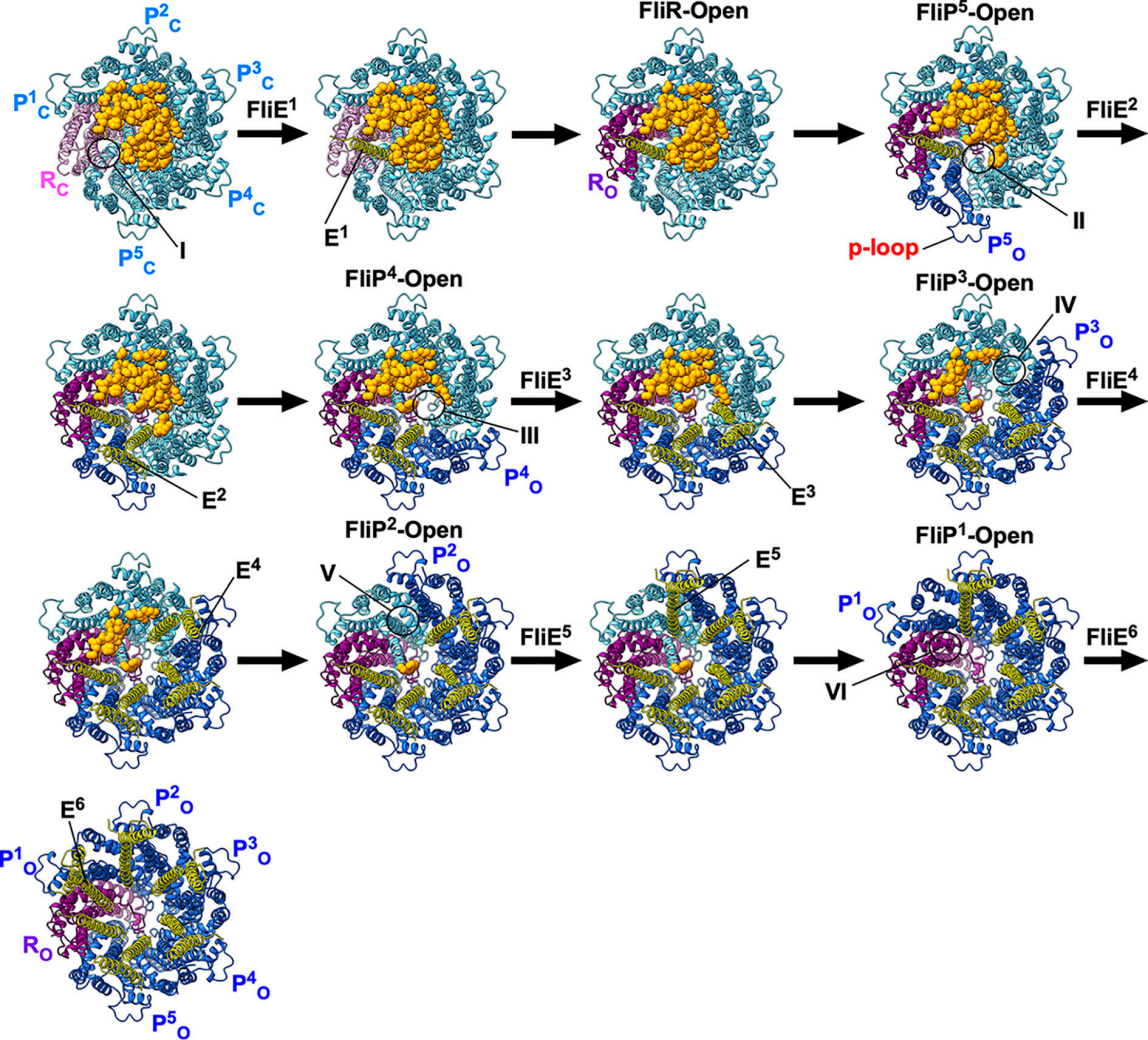
FliE assembly mechanism. The periplasmic gate of the FliPQR complex is completely closed by the β-cap while leaving a narrow pore (I), as shown in the CPK representation. The first FliE subunit (E^1^) is transported into the export channel by the fT3SS, and α3 of FliE^1^ inserts into the narrow pore of the β-cap. The interaction between the closed form of FliR (R_C_) and FliE^1^ induces a conformational change of α1 of FliR, allowing this helix not only to adopt an open conformation (R_O_) but also to form the D0-like domain together with α3 of FliE^1^ (*SI Appendix*, Fig. S14). Then, α3 of FliE^1^ associates with the closed form of the FliP^5^ subunit (P^5^_C_), thereby inducing a conformational change of the conserved MSTF motif of FliP (*SI Appendix*, Fig. S15). As a result, FliP^5^ adopts an open conformation (P^5^_O_), creating the next insertion site (II) for the second FliE subunit (FliE^2^) within the β-cap. This open conformation is stabilized by the direct contact of the p-loop of FliP^5^_O_ with the inner wall of the MS-ring. When FliE^2^ inserts into this site, helices α2 and α3 of FliE^2^ form domain D0 between FliP^5^_O_ and the closed form of FliP^4^ (P^4^_C_). The FliE^2^–FliP^4^_C_ interaction allows FliP^4^_C_ to adopt the open conformation (P^4^_O_). The FliE assembly proceeds by repeating these steps. When the sixth FliE subunit (FliE^6^) inserts between FliP^1^_O_ and FliR_O_, the periplasmic gate of the FliPQR complex is completely opened, allowing other flagellar structural subunits to go through its channel, diffuse through the central channel of the growing flagellum, and assemble at the distal end (Movie S2).

How does FliE gain access to the distal face of the FliPQR complex? The β-cap tightly seals the periplasmic gate, but a narrow pore remains open between FliR and FliP^5^ that is wide enough to permit the passage of a single α-helix (Fig. 2*C*). Helix α1 of FliP^1^ projects toward the β-hairpins of FliP^4^ and FliP^5^ in the closed form of the FliPQR complex, and its Trp-38 residue makes direct contact with Trp-38 of FliP^4^ and FliP^5^ (Fig. 2*B*). As a result, helix α1 of FliP^1^ efficiently and precisely directs the N-terminal helix of FliE^1^, which contains the export signal recognized by the fT3SS, into the pore. Once secreted, FliE^1^ initiates the assembly of FliE subunits on the scaffold provided by the β-cap, which then leads to the disruption of the β-cap and the conformational changes in FliP and FliR that generate the open form of the gate (Fig. 7 and Movie S2).

In the remaining five FliE subunits, the α2 and α3 helices form domain D0, the inner core domain of the flagellar axial structure (12, 13). Because of steric hindrances between α2 of FliE^1^ and α1 of FliR, α3 of FliE^1^ binds to α1 of FliR, thereby forming the D0-like domain (*SI Appendix*, Fig. S14 *A* and *B*). Because steric hindrance also occurs between these two α-helices, α1 of FliR moves outward by removing the kink at Tyr-16 (*SI Appendix*, Fig. S14*C*), thereby detaching β1 of FliR from the β-cap. Consequently, FliR adopts the open form (Fig. 7, step 3), and the FliE^1^–FliR interaction is stabilized by α1 of FliP^1^ (*SI Appendix*, Fig. S15*A*) until the sixth FliE subunit is inserted between FliP^1^ and FliR.

The FliE^1^–FliR interaction also allows α3 of FliE^1^ to bind to α1 of FliP^5^. There is steric hindrance between these two α-helices, but Met-102 of FliE^1^ interacts with Leu-90 of FliP^5^ (*SI Appendix*, Fig. S15 *B* and *C*) to induce a structural remodeling of the hydrophobic interaction networks surrounding the MTSF motif. As a result, both α1 and HTH_α3-α4_ of FliP^5^ move outward, not only dislodging its β-hairpin from the β-cap but also creating the insertion site for the second FliE subunit (FliE^2^) within the cap (Fig. 7, step 4). The p-loop of FliP^5^ associates with the inner wall of the MS-ring, stabilizing the open conformation of FliP^5^. When FliE^2^ inserts into the second site (step 5), α3 of FliE^2^ interacts with α1 of FliP^4^, which allows FliP^4^ to adopt the open conformation and creates the third FliE insertion site within the β-cap (step 6). This interaction also allows α2 and α3 of FliE^2^ to form the D0 domain. By repeating this process, FliE assembly proceeds in the counterclockwise direction when viewed from the periplasmic side. When the sixth FliE subunit inserts between FliP^1^ and FliR, the periplasmic gate of the FliPQR complex is completely open. Although our model of FliE assembly on the FliPQR complex is consistent with available biochemical and structural data, direct structural evidence of a FliPQR–FliE complex is currently lacking. Future studies will be necessary to confirm this interaction at high resolution.

In summary, the β-cap seals the periplasmic gate of the export channel to prevent premature export of flagellar proteins by the fT3SS until FliE assembles at the tip of the FliPQR complex to serve as the template for rod assembly. The β-cap also serves as a scaffold for FliE assembly. The interactions of FliE with FliP and FliR induce remodeling of the hydrophobic networks around the conserved MTSF motif, allowing the periplasmic gate, initially closed like a floral bud, to open like a blooming flower. Thus, the β-cap enables FliE to couple the opening of the periplasmic gate with its efficient and robust assembly on the top of FliP and FliR.

## Materials and Methods

*Salmonella* strains and plasmids used in this study are listed in *SI Appendix*, Table S6. The His-tagged FliPQR complex, solubilized with LMNG, was purified by Ni affinity and size-exclusion chromatography and reconstituted into a peptidisc. Structural analysis was performed by cryoEM single particle reconstruction, and model refinement statistics are provided in *SI Appendix*, Table S1. Structural comparisons and BSA calculations were conducted using ChimeraX and PDBePISA, respectively. Flagella-driven motility and fT3SS-mediated protein secretion were assessed by soft agar motility assay and immunoblotting, respectively. Detailed experimental procedures are provided in *SI Appendix*.

## Supplementary Material

Appendix 01 (PDF)

Movie S1. Structural comparison between the closed and open forms of the FliPQR export channel complex.

Movie S2. FliE assembly mechanism.

## Data Availability

The cryoEM map and atomic model of the *Salmonella* FliPQR complex reconstituted in a peptidisc have been deposited in the Electron Microscopy Data Bank under an accession code EMD-61993 (46) and the Protein Data Bank under an accession code 9K29 (47). All other data are included in the manuscript and/or supporting information.
