# Supplementary material for: A β-cap on the FliPQR protein-export channel acts as the cap for initial flagellar rod assembly
Source: Proc Natl Acad Sci U S A. 2025 Aug 20;122(34):e2507221122. doi: 10.1073/pnas.2507221122 (PMC12403130; doi:10.1073/pnas.2507221122)
Supplement: Supplementary file 1 — Appendix 01 (PDF) [file pnas.2507221122.sapp.pdf]

PNAS

Supporting Information for
A β-cap on the FliPQR protein-export channel acts as the cap for initial flagellar rod assembly

Miki Kinoshita[a,b], Tomoko Miyata[a,b], Fumiaki Makino[a,b,c], Katsumi Imada[d], Keiichi Namba[a,b,*], Tohru Minamino[a,*]

* Keiichi Namba
Email: namba.keiichi.fbs@osaka-u.ac.jp

* Tohru Minamino
Email: minamino.tohru.fbs@osaka-u.ac.jp

This PDF file includes:

Supporting text
Figures S1 to S15
Tables S1 to S6
Legends for Movies S1 to S2
SI References

Other supporting materials for this manuscript include the following:

Movies S1 to S2

Supporting Information Text

Materials and Methods

Bacterial strains, plasmids, and DNA manipulations. *Salmonella* strains and plasmids used in this study are listed in *SI appendix*, Table S6. DNA manipulations were performed using standard protocols. Site-directed mutagenesis was carried out using Prime STAR Max Premix as described in the manufacturer's instructions (Takara Bio). All *fliP* and *fliR* mutations were confirmed by DNA sequencing (Eurofins Genomics).

Purification of the His-tagged FliPQR complex (FliPQR-His). A 13 ml of overnight culture of *Salmonella* SJW1368 cells carrying pMKM10001 (pTrcCES3/ FliP + FliQ + FliR-His) were inoculated into a 1.3 l of fresh 2×YT [1.6% (w/v) Bacto-tryptone, 1.0% (w/v) Bacto-yeast extract, 0.5% (w/v) NaCl] containing 100 µg/ml ampicillin. The cells were grown at 30ºC until the cell density had reached an OD_{600} of about 0.5–1.0, followed by the incubation at 16ºC for another 24h. *Salmonella* cells were harvested by centrifugation (6,400 g, 10min, 4ºC) and stored at -80ºC. The cells were thawed, resuspended in 20 mM Tris-HCl, pH 8.0, 3 mM EDTA, and disrupted by sonication. After centrifugation (20,000 g, 15 min, 4ºC) to remove cell debris, cell lysates were ultracentrifuged (110,000 g, 1 h, 4ºC). Harvested membranes were solubilized in 50 mM Tris-HCl, pH 8.0, 300 mM NaCl, 5% (w/v) glycerol, 20 mM imidazole, 1% (w/v) LMNG at 4ºC for 1 h and ultracentrifuged (110.000g, 1h, 4ºC) to remove the insoluble membranes. Solubilized membrane proteins were loaded onto a Ni-NTA agarose column (QIAGEN) and washed extensively with 50 mM Tris-HCl, pH 8.0, 300 mM NaCl, 5% (w/v) glycerol, 20 mM imidazole, and 0.01% (w/v) LMNG. Proteins were eluted with a 100-400 mM imidazole gradient. Fractions containing FliPQR-His were concentrated, followed by size exclusion chromatography (SEC) with a Superdex 200 10/300 column (GE Healthcare) equilibrated with 20 mM Tris-HCl, pH 8.0, 300 mM NaCl, 1 mM EDTA, 5 %(w/v) glycerol, 0.005% (w/v) LMNG.

Reconstitution of the FliPQR-His into peptidisc. Fractions containing FliPQR-His at the highest concentrations eluted from the SEC column were selected and used for reconstitution of FliPQR-His into peptidisc. The peptidisc solution was prepared from bulk lyophilized peptidisc (Peptidisc Biotech, Vancouver, BC, Canada) dissolved in 20 mM Tris-HCl, pH 7.8 to a final concentration of 5 mg/ml. Dissolved peptidisc was mixed with the solubilized FliPQR-His sample in 1:1 weight ratio and incubated at room temperature for 30 min. Then, the mixture was flowed through the Superdex 200 10/300 column equilibrated with 20 mM Tris-HCl, pH 8.0, 150mM NaCl to obtain FliPQR-His in detergent-free solution.

Negative-staining electron microscopy. A 3 µl aliquot of FliPQR-His was applied to a thin carbon-coated copper grid that had been glow-discharged for 20 s. Excess liquid was removed with filter paper, and the sample was stained with 2% (w/v) uranyl acetate. Grids were air-dried for at least 30 min at room temperature. Electron micrographs were acquired using a JEM-1400Flash (JEOL, Tokyo, Japan) operating at an accelerating voltage of 100 kV and recorded at a magnification of x100,000.

Sample preparation and cryoEM data collection. Fluorinated Fos-Choline was added to the purified FliPQR-His solution at a final concentration of 0, 1.0, or 2.0 mM before grid preparation. A 2.7 µl aliquot of the sample solution (2.0 mg/ml) were applied onto a glow-discharged holey carbon-coated grid (Quantifoil 200mesh, Cu R1.2/1.3). The grid was blotted by a filter paper at 4ºC for 3 sec and quickly frozen in liquid ethane using a Vitrobot Mark IV system (Thermo Fisher Scientific, 4°C and 100% humidity).

The grids were inserted into a CRYO ARM 300 transmission electron microscopy (JEOL Ltd. Japan) equipped with a cold field-emission electron gun operated at 300 kV and an Ω-type energy filter with a 20 eV slit width. CryoEM images were recorded with a K3 direct electron detector camera (Gatan, USA) at a nominal magnification of ×60,000, corresponding to an image pixel size

of 0.87 Å, using SerialEM (1). The holes were detected using YoneoLocr (2). Movie frames were recorded in CDS counting mode with a total exposure time of 3 sec and a total dose of ~40 electrons $Å^{-2}$. Each movie was fractionated into 40 frames. In total, 13,230 movies were collected.

CryoEM image processing. Single particle analysis was performed using RELION 3.1 (3). Image processing procedure is described in *SI appendix*, Fig. S2B. After performing motion corrections to align all micrographs, followed by the estimation of parameters of the contrast transfer fraction (CTF), particle images were automatically selected via LoG auto-picking, and the selected particles were extracted into a box of 256 × 256 pixels (1,505,398 particles). Particle images from high-quality 2D class averages were used to generate an initial 3D model, followed by classification into three distinct three classes. In total, 164,019 particles were subjected to 3D classification with C1 symmetry into two classes. After 3D refinement for the better class, postprocessing yielded a 3D map at a resolution of 3.0 Å (109,333 particles), based on the 0.143 criterion of the Fourier shell correlation (FSC) (*SI appendix*, Fig. S2C). The cryoEM density map was deposited into Electron Microscopy Data Bank with an accession code EMD-61993.

Model building and refinement of the FliPQR complex. The atomic model of the FliPQR complex was constructed using Coot (4). PHENIX was used for real-space refinement based on the cryoEM map (5). Summary of model refinement and statistics are described in *SI appendix*, Table S1. Structural comparison and analyses were conducted using UCSF ChimeraX (6). The buried surface area (BSA) of the conserved Leu-92 residue in both the open and closed conformations of the FliPQR complex were calculated using PDBePISA (Proteins, Interfaces, Structures and Assemblies) (https://www.ebi.ac.uk/pdbe/pisa/). The atomic coordinates have been deposited in the Protein Data Bank with an accession code 9K29.

Motility assay in soft agar. Fresh colonies were inoculated into soft agar plates [1% (w/v) tryptone, 0.5% (w/v) NaCl, 0.35% Bacto agar] containing 100 µg/ml ampicillin and incubated at 30°C. At least five independent measurements were performed.

Secretion assay. A 100 µl of the overnight culture of *Salmonella* cells was inoculated into a 5 ml of fresh L-broth [1% (w/v) tryptone, 0.5% (w/v) yeast extract, 0.5% (w/v) NaCl] containing 100 µg/ml ampicillin and incubated at 30 ºC with shaking until the cell density had reached an OD_{600} of ca. 1.4–1.6. Cultures were centrifuged to obtain cell pellets and culture supernatants, separately. The cell pellets were resuspended in sodium dodecyl sulfate (SDS)-loading buffer solution [62.5 mM Tris-HCl, pH 6.8, 2% (w/v) SDS, 10% (w/v) glycerol, 0.001% (w/v) bromophenol blue] containing 1 µl of 2-mercaptoethanol. Proteins in each culture supernatant were precipitated by 10% trichloroacetic acid and suspended in a Tris/SDS loading buffer (one volume of 1 M Tris, nine volumes of 1 X SDS-loading buffer solution) containing 1 µl of 2-mercaptoethanol. Both whole cellular proteins and culture supernatants were normalized to a cell density of each culture to give a constant cell number. After boiling at 95ºC for 3 min, the samples were separated by sodium dodecyl sulfate-polyacrylamide gel electrophoresis (SDS-PAGE) and transferred to a nitrocellulose membrane (Bio-Rad) using a transblotting apparatus (Hoefer). Then, immunoblotting with polyclonal anti-FlgD or anti-FliP antibody as the primary antibody and anti-rabbit IgG, HRP-linked whole Ab Donkey (GE Healthcare) as the secondary antibody was carried out using iBand Flex Western Device as described in the manufacturer's instructions (Thermo Fisher Scientific). Detection was performed with Amersham ECL Prime western blotting detection reagent (Cytiva). Chemiluminescence signals were captured by a Luminoimage analyzer LAS-3000 (GE Healthcare). Bands of prestained protein molecular weight markers (Bio-Rad) transferred to each membrane were also photographed with the LAS-3000 under brightfield illumination and combined with each immunoblot image to identify the band of interest. All image data were processed with Photoshop software (Adobe). At least three independent experiments were performed.

Purification of the His-tagged FliPQR complex with the *fliP(L92A)* mutation. A 13 ml of overnight culture of *Salmonella* SJW1368 cells carrying pMKM10001(L92A) were inoculated into a 1.3 l of fresh 2×YT containing 100 µg/ml ampicillin. The cells were grown at 30ºC until the cell density had reached an OD_{600} of about 0.5–1.0, followed by the incubation at 16ºC for another 24h.

The cells were harvested by centrifugation (6,400 g, 10min, 4ºC) and stored at -80ºC. The cells were thawed, resuspended in 20 mM Tris-HCl, pH 8.0, 3 mM EDTA, and disrupted by sonication. After centrifugation (20,000 g, 15 min, 4ºC) to remove cell debris, cell lysates were ultracentrifuged (110,000 g, 1 h, 4ºC). The harvested membranes were solubilized in 50 mM Tris-HCl, pH 8.0, 300 mM NaCl, 5% (w/v) glycerol, 20 mM imidazole, 1% (w/v) LMNG at 4ºC for 1 h and ultracentrifuged (110.000g, 1h, 4ºC) to remove the insoluble membranes. Solubilized membrane proteins were loaded onto a Ni-NTA agarose column and washed extensively with 50 mM Tris-HCl, pH 8.0, 300 mM NaCl, 5% (w/v) glycerol, 20 mM imidazole, and 0.01% (w/v) LMNG. Proteins were eluted with a 100-400 mM imidazole gradient. Fractions containing FliP(L92A)QR-His were concentrated, followed by size exclusion chromatography (SEC) with a Superdex 200 10/300 column equilibrated with 20 mM Tris-HCl, pH 8.0, 300 mM NaCl, 1 mM EDTA, 5 %(w/v) glycerol, 0.005% (w/v) LMNG.

Multiple sequence alignment. Multiple sequence alignment was carried out using Clustal Omega (https://www.ebi.ac.uk/jdispatcher/msa/clustalo) (7). Evolutionarily conserved residues of FliP and FliR were also analyzed using the ConSurf web server (https://consurf.tau.ac.il/consurf_index.php) (8).

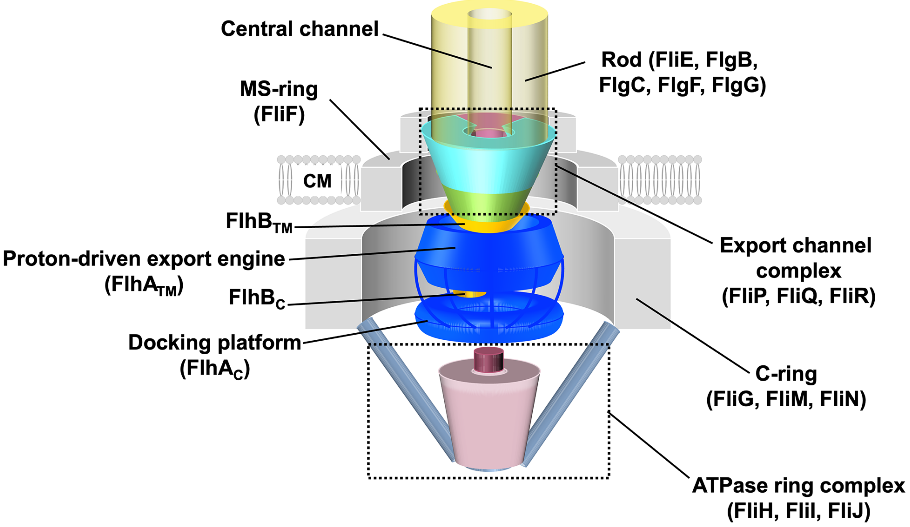

Fig. S1. Schematic diagram of the flagellar type III secretion system. The flagellar type III secretion system consists of a transmembrane export gate complex made up of FlhA, FlhB, FliP, FliQ, and FliR and a cytoplasmic ATPase ring complex consisting of FliH, FliI, and FliJ. The export gate complex is located within the central pore of the basal body MS-ring, whereas the cytoplasmic ATPase firmly associates with the basal body C-ring through interactions between FliH and FliN. FliP, FliQ, and FliR form an export channel complex for efficient and rapid export of flagellar structural subunits. FlhB associates with the FliPQR complex and plays an important role in opening and closing the cytoplasmic gate of the channel. The N-terminal transmembrane domains of FlhA serve as a proton-driven export engine that couples inward-directed proton flow through the proton channel with outward-directed protein translocation through the export channel. The C-terminal domains of FlhA (FlhA$_{C}$) and FlhB (FlhB$_{C}$) project into the central cavity of the C-ring and act as a substrate docking platform. Because the FliPQR complex has a helical symmetry, FliE and FlgB can directly assemble onto FliP and FliR to form the most proximal part of the rod. CM, cytoplasmic membrane.

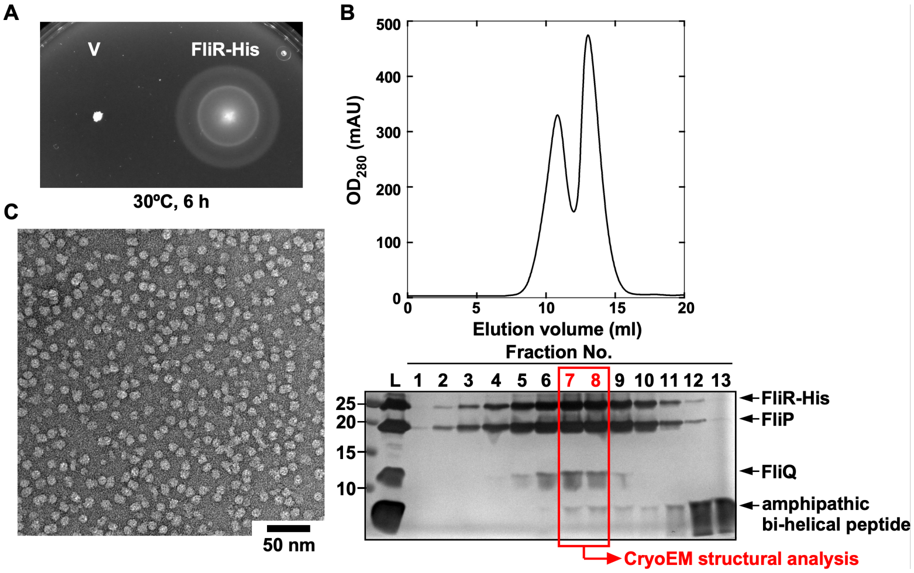

Fig. S2. Reconstitution of the FliPQR-His complex into peptidisc. (A) Effect of His-tag attachment to FliR on flagella-mediated motility. Motility of a *Salmonella fliR* null mutant harboring pBAD24E (V) or pMKM701 (FliR-His) in soft agar. The plates were incubated at 30ºC for 6 hours. At least seven independent assays were carried out. **(B)** Elution profile of the FliPQR-His complex reconstituted into peptidisc formed by amphipathic bi-helical peptides, using a Superdex 200 10/300 column equilibrated with 20 mM Tris-HCl, pH 8.0, 150mM NaCl (upper panel). Elution fractions were analyzed by SDS-PAGE, followed by silver staining (lower panel). Positions of molecular mass markers (kDa) are indicated on the left. **(C)** Negative-stain EM image of purified FliPQR-His complex reconstituted into peptidisc. Fractions 7 and 8 were collected, concentrated, and negatively stained with 2% (w/v) uranyl acetate. Electron micrographs were recorded at a magnification of x100,000.

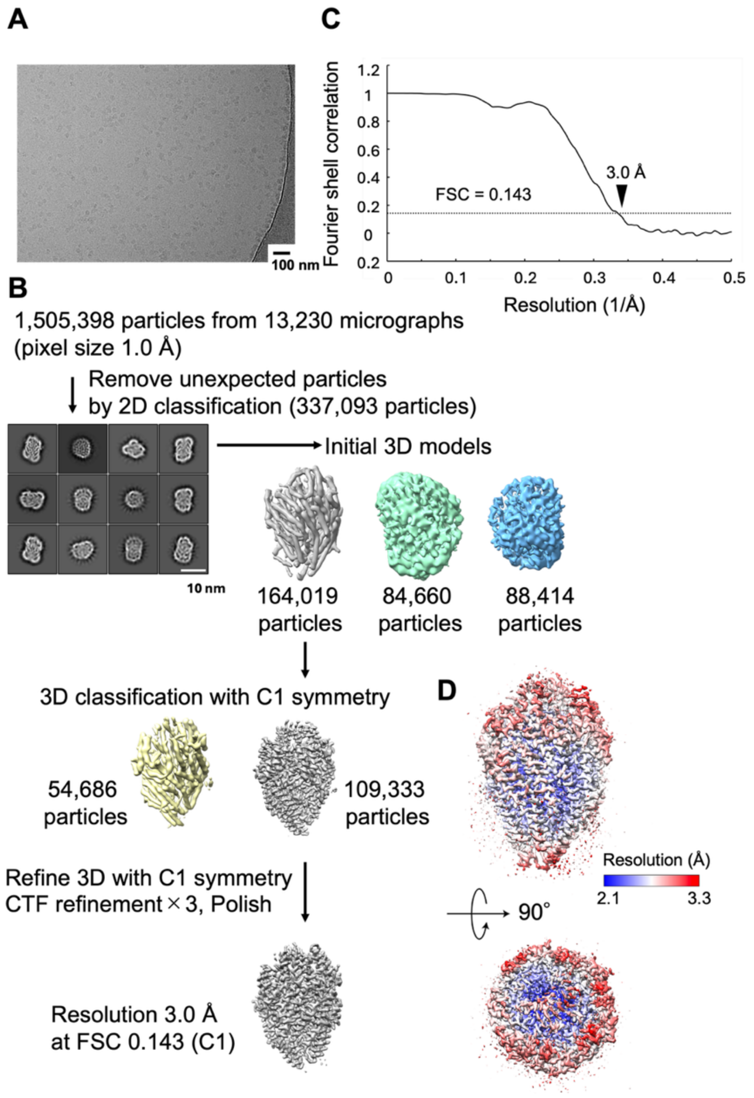

Fig. S3. CryoEM single-particle 3D image analysis of the FliPQR complex reconstituted in a peptidisc. (A) Representative cryoEM micrograph of the FliPQR complex reconstituted in a peptidisc. **(B)** Workflow of the cryoEM single-particle 3D reconstruction. **(C)** Fourier shell correlation (FSC) curve for the final density map of the FliPQR complex (EMDB ID: EMD-61993). **(D)** Local resolution map of the FliPQR complex, colored from blue (2.1 Å) to red (3.3 Å).

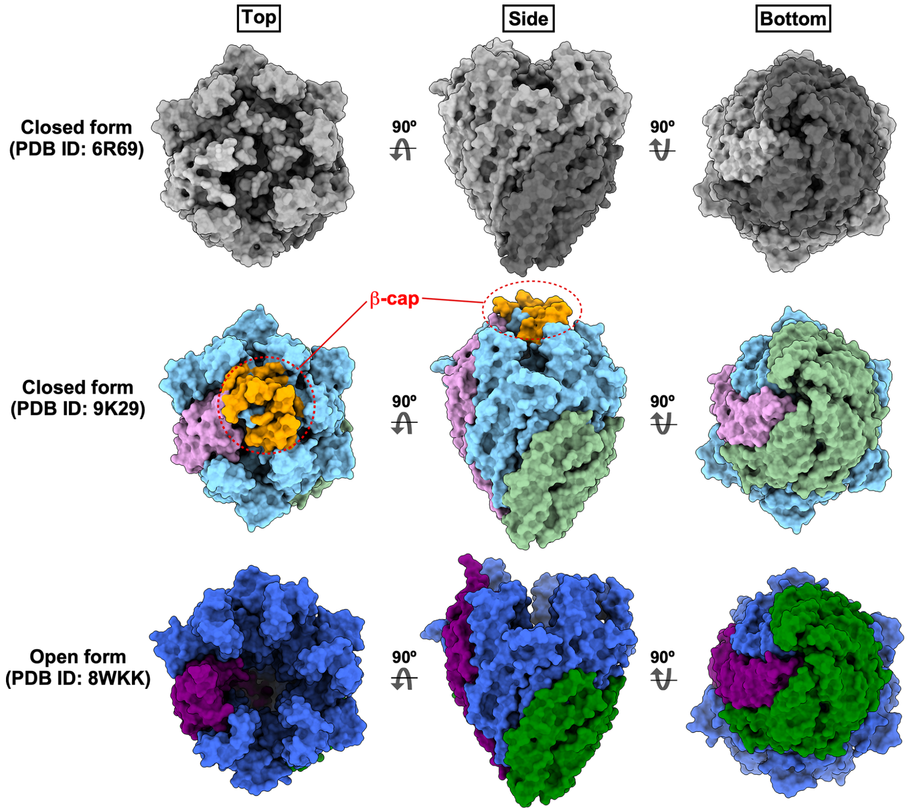

Fig. S4. Surface maps of three different cryoEM structures of the FliPQR complex. The periplasmic gate is fully closed by the β-cap in the 9K29 structure (middle panels). In contrast, the β-cap is missing in the 6R69 (top panels) and 8WKK (lower panels) structures. The cytoplasmic gate is closed in these three different structures.

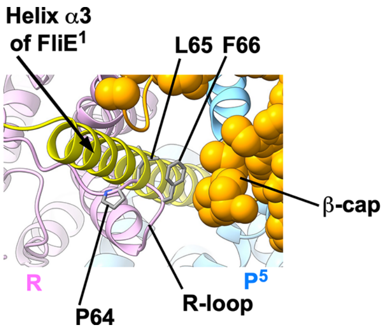

Fig. S5. Steric clash between the R-loop of FliR and helix α3 of the first FliE subunit. The FliR subunit in the 9K29 structure is superimposed onto the equivalent coordinate of the 8WKK structure. Residues 64–66 of FliR in the R-loop (plum), which is formed by residues 55–68, exhibits an apparent steric clash with helix α3 of the first FliE subunit (FliE1, yellow). This FliE helix cannot be fully accommodated without local accommodation.

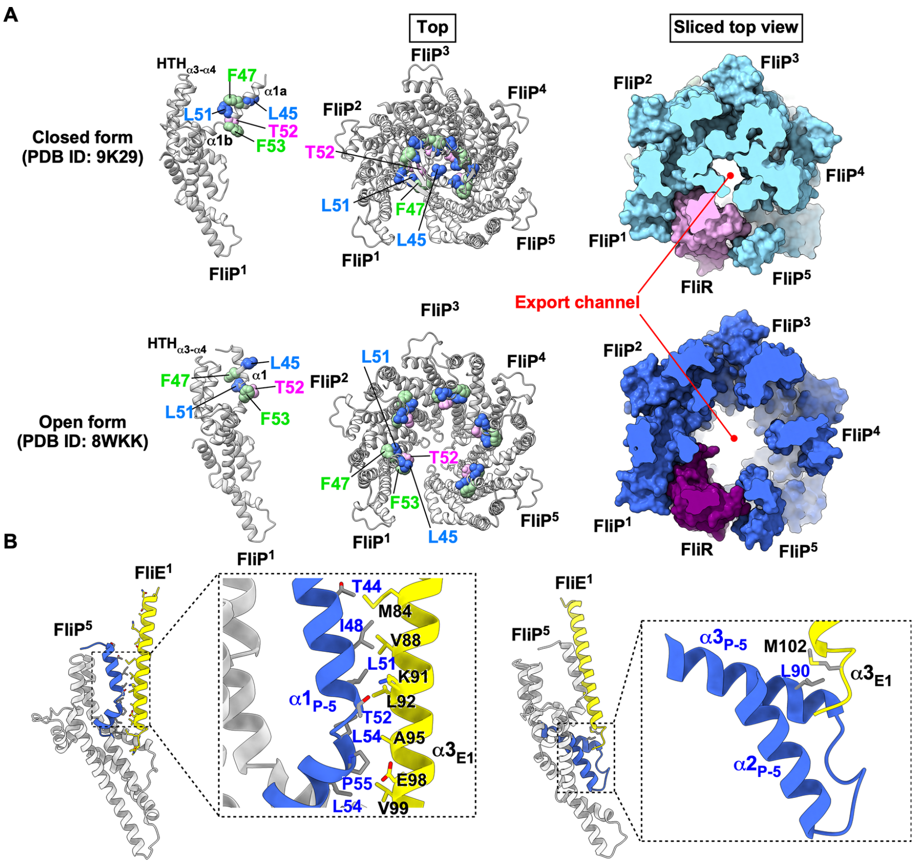

Fig. S6. Dual roles of helix α1 of FliP in flagellar protein export and assembly. (A) Location of conserved Leu-45, Phe-47, Leu-51, Thr-52, and Phe-53 residues in the N-terminal α1 helix of FliP. Leu-45, Phe-47, Leu-51, Thr-52, and Phe-53 are conserved among FliP homologues. These residues are in helix α1a of FliP and form a hydrophobic core in the closed FliPQR complex (PDB ID: 9K29). These residues are also involved in the interaction with FliE in the open structure (PDB ID: 8WKK). The right panels show the horizontally sliced top views of the surface maps. The periplasmic gate is closed by hydrophobic core formation by the N-terminal α1 helices of the five FliP subunits in the closed structure. **(B)** Interaction between FliP and FliE in the 8WKK structure. Helix α3 of the first FliE subunit ($FliE^1$) firmly associates with helix α1 of the fifth FliP subunit ($FliP^5$). The C-terminal end of $FliE^1$ also binds to the helix-turn-helix structure formed by helices α2 and α3 of $FliP^5$. Leu-90 of $FliP^5$ makes hydrophobic contact with Met-102 of $FliE^1$.

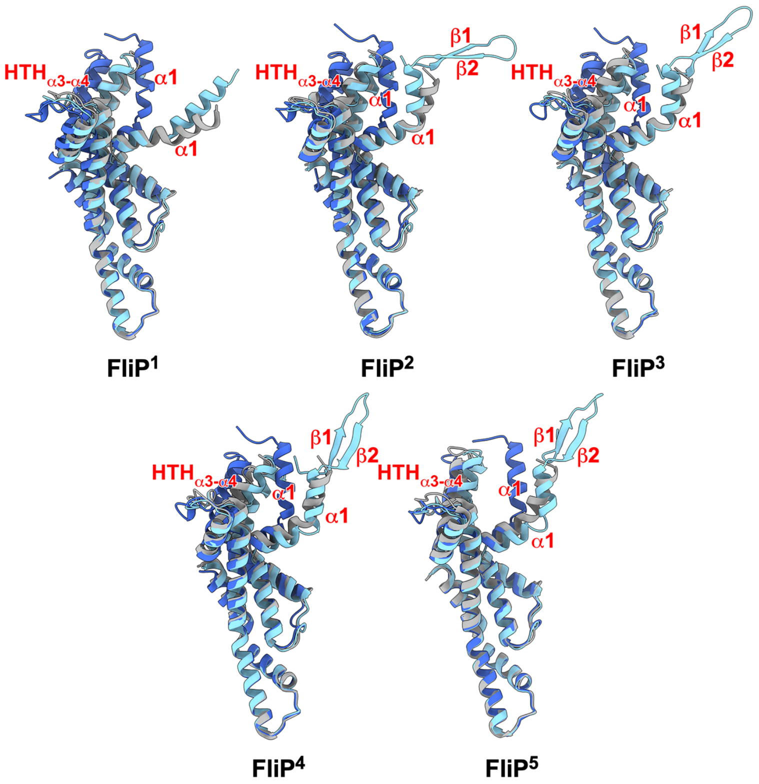

(6R69, dark gray; 9K29, sky blue; 8WKK, royal blue)

Fig. S7. Structural comparisons of each FliP subunit in the FliPQR complex of the present study (PDB ID: 9K29) with those of previous studies.. Each FliP subunit from the 6R69 (dark gray) and 8WKK (royal blue) structures was individually superimposed onto the corresponding subunit from the 9K29 structure (sky blue). The FliP subunit located at the top of the right-handed helical structure of the FliPQR complex is designated as the first FliP subunit ($FliP^1$). The remaining subunits, arranged along the helical staircase, are subsequently referred to as the second ($FliP^2$), third ($FliP^3$), fourth ($FliP^4$), and fifth ($FliP^5$) subunits, respectively. The root mean square deviations (RMSDs) for these superpositions are provided in *SI Appendix*, Table S3.

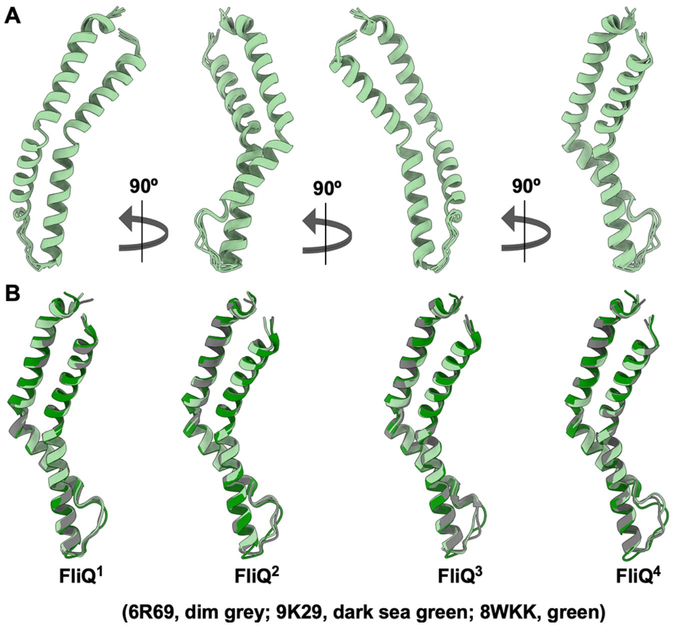

Fig. S8. Structural comparisons of FliQ subunits in the FliPQR complex. (A) Structural alignment of the four FliQ subunits in the 9K29 structure. The root mean square deviations (RMSDs) for these superpositions are provided in *SI Appendix*, Table S4. **(B)** Superposition of each FliQ subunit obtained in this study (PDB ID: 9K29; dark sea green) with the corresponding subunits from the 6R69 (dim gray) and 8WKK (green) structures. The FliQ subunit that directly associates with FliR is designated as the first FliQ subunit ($FliQ^1$). The remaining subunits, arranged along the helical staircase, are subsequently referred to as the second ($FliQ^2$), third ($FliQ^3$), and fourth ($FliQ^4$) subunits. Each FliQ subunit from the 6R69 and 8WKK structures was individually superimposed onto its corresponding subunit in the 9K29 structure. The RMSD values are listed in *SI Appendix*, Table S3.

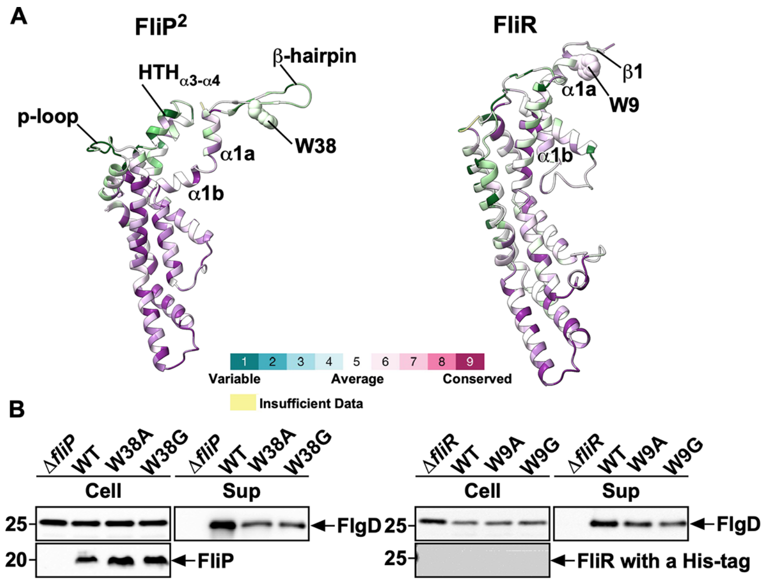

Fig. S9. Mutational analysis of Trp-38 of FliP and Trp-9 of FliR. (A) Evolutionarily conserved residues of FliP (left panel) and FliR (right panel). Conservation scores were calculated using the ConSurf web server. Residues are colored according to conservation among FliP and FliR amino acid sequences derived from 150 bacterial species. **(B)** Flagellar protein secretion assay. Whole-cell (Cell) and culture supernatant (Sup) fractions were prepared from a *fliP* null mutant (left panel) harboring pTrc99AFF4 (Δ*fliP*), pKY69 (WT), pMKM69(W38A) (W38A), or pMKM69(W38G) (W38G); and a *fliR* null mutant (right panel) carrying pBAD24E (Δ*fliR*), pMKM701 (WT), pMKM701(W9A) (W9A), or pMKM701(W9G) (W9G). A 5 µl aliquot of each protein sample, normalized to OD_{600}, was subjected to SDS-PAGE and analyzed by immunoblotting using polyclonal anti-FlgD, polyclonal anti-FliP, and monoclonal anti-His antibodies. Molecular mass markers (kDa) are indicated on the left. At least three independent experiments were performed.

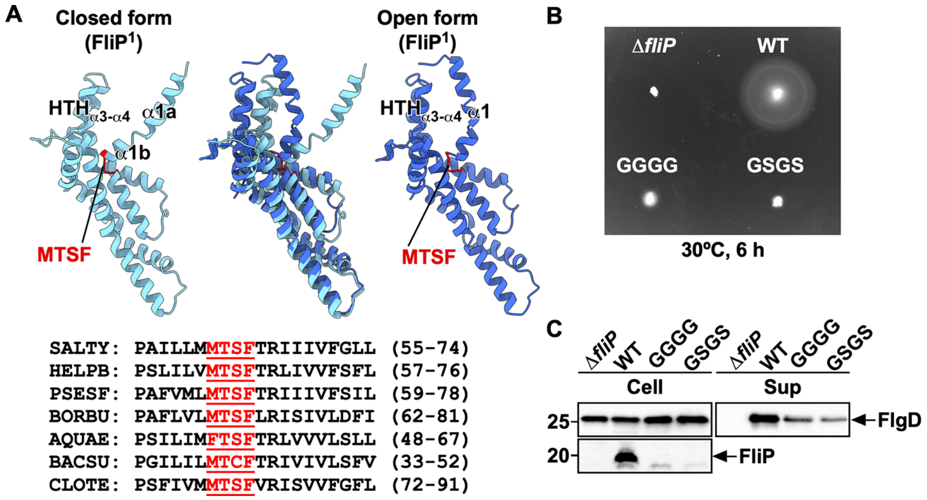

Fig. S10. Mutational analysis of the conserved MTSF motif in FliP. (A) Structural comparison and multiple sequence alignments of the conserved MTSF motif of FliP. The closed (PDB ID: 9K29, sky blue) and open (PDB ID: 8WKK, royal blue) forms of the first FliP subunit ($FliP^1$) are shown (See Movie S1). Multiple sequence alignment was carried out by Clustal Omega. The conserved MTSF motif of FliP is highlighted in red. UniProt Accession numbers: *Salmonella enterica* (SALTY), C7BZE6; *Helicobacter pylori* (HELPB), C7BZE6; *Pseudomonas syringae* (PSESF), A0A2V0QEJ7; *Borreliella burgdorferi* (BORBU), Q44763; *Aquifex aeolicus* (AQUAE), O67750; *Bacillus subtilis* (BACSU), P35528; *Clostridium tetani* (CLOTE), Q893Z3. **(B)** Motility of a *Salmonella fliP* null mutant carrying pTrc99AFF4 (indicated as Δ*fliP*), pKY69 (indicated as WT), pMKM69-G4 (indicated as GGGG), and pMKM69-GS2 (indicated as GSGS) in soft agar. Plates were incubated at 30ºC for 6 hours. At least seven independent assays were performed. **(C)** Flagellar protein secretion assay. Whole-cell (Cell) and culture supernatant (Sup) fractions were prepared from the above transformants. A 5 μl solution of each protein sample, normalized to OD_{600}, was subjected to SDS-PAGE, followed by immunoblotting with polyclonal anti-FlgD (first row) and anti-FliP (second row) antibodies. Molecular mass markers (kDa) are shown on the left. At least three independent assays were carried out.

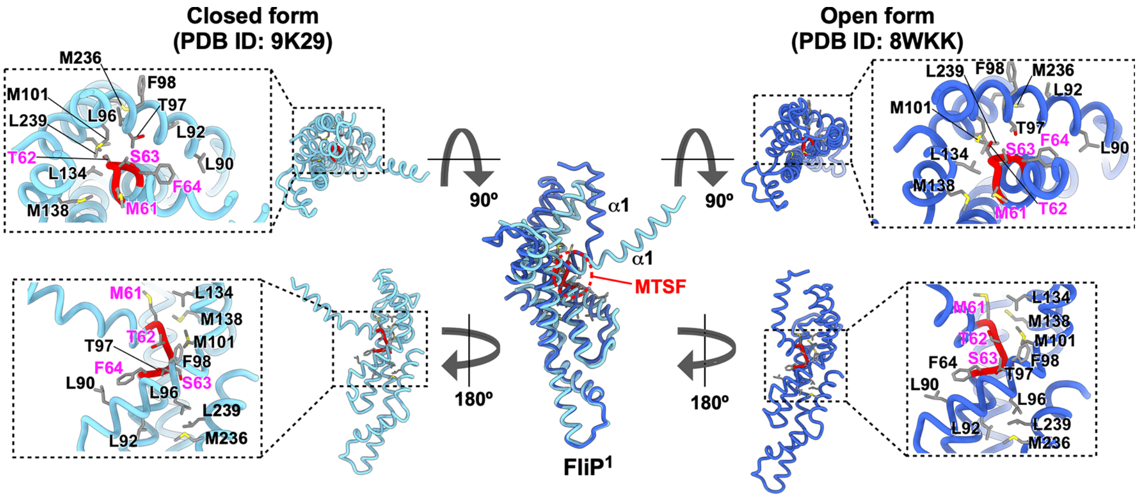

Fig. S11. Hydrophobic interaction networks surrounding the MTSF motif (red) in the closed (PDB ID: 9K29) and open (PDB ID: 8WKK) forms of FliP.

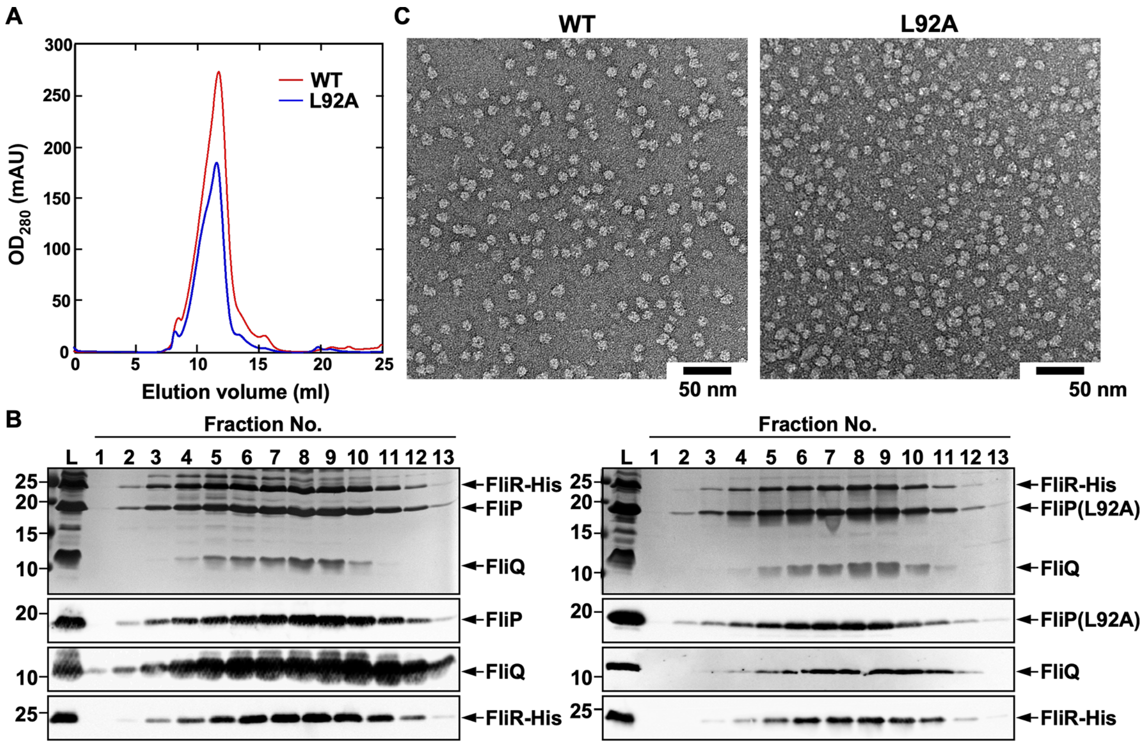

Fig. S12. Effect of the FliP(L92A) substitution on FliPQR complex formation. (A, B) Elution profiles of the wild-type FliPQR complex (WT, red line) and the FliP(L92A)QR variant (L92A, blue line) obtained using a Superdex 200 10/300 column equilibrated with 20 mM Tris-HCl, pH 8.0, 300 mM NaCl, 1 mM EDTA, 5% Glycerol, 0.005% LMNG **(A)**. Elution fractions were analyzed by SDS-PAGE, followed by silver staining (first row) and immunoblotting with polyclonal anti-FliP (second row), polyclonal anti-FliQ (third row), and monoclonal anti-His (fourth row) antibodies **(B)**. Molecular mass markers (kDa) are indicated on the left. **(C)** Negative-stain EM images of the FliPQR complex with or without the L92A substitution. Electron micrographs were recorded at a magnification of x100,000.

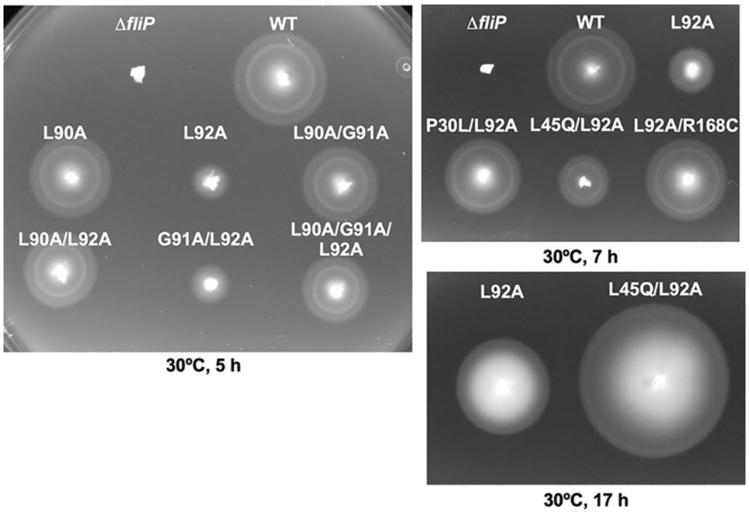

Fig. S13. Isolation of up-motile mutants from the *fliP(L92A)* mutant in soft agar. Motility of a *Salmonella fliP* null mutant carrying pTrc99AFF4 (indicated as Δ*fliP*), pKY69 (indicated as WT), , pMKM69(L90A) (indicated as L90A), pMKM69(L92A) (L92A), pMKM69(L90A/G91A) (L90A/G91A), pMKM69(L90A/L92A) (L90A/L92A), pMKM69(G91A/L92A) (G91A/L92A), pMKM69(L90A/G91A/L92A) (L90A/G91A/L92A), pMKM69(L92A)-SP1 (indicated as P30L/L92A), pMKM69(L92A)-SP2 (indicated as L45Q/L92A), and pMKM69(L92A)-SP3 (indicated as L92A/R168C) in soft agar. Plates were incubated at 30ºC. At least seven independent assays were carried out.

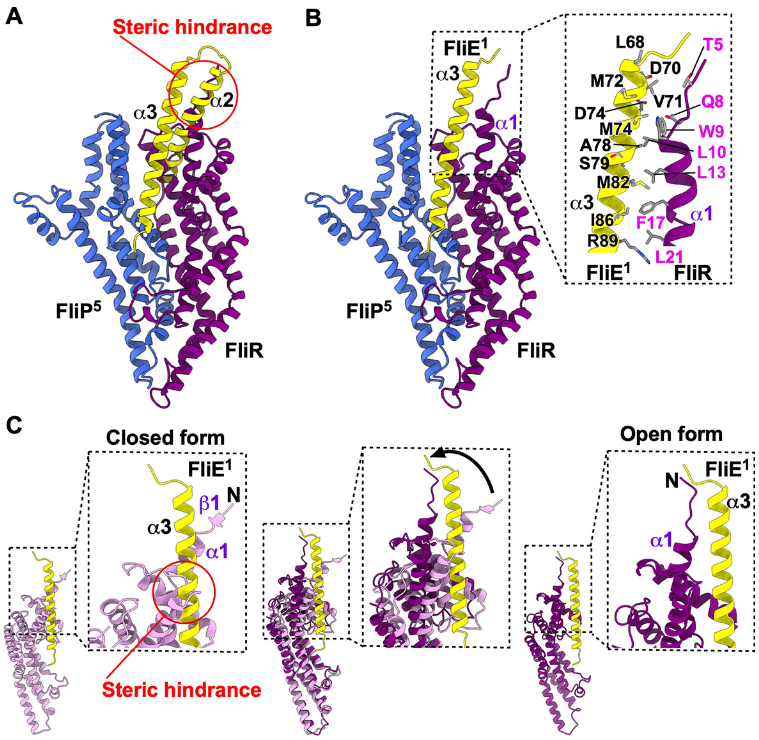

Fig. S14. Interaction between the first FliE subunit and FliR. (A) Interaction between domain D0 of FliE and FliR. FliE (yellow) contains three α-helices with the α2 and α3 helices forming domain D0. The D0 domain is superimposed onto α3 of the first FliE subunit (FliE^1) in the open structure of the FliPQR complex (PDB ID: 8WKK). Because α2 of the D0 domain collides with α1 of FliR at the position indicated by the red circle, α2 of FliE^1 cannot form domain D0 along with α3. Consequently, α2 of FliE^1 is invisible in the 8WKK structure. **(B)** Interaction between α3 of FliE^1 and α1 of the open form of FliR (purple). When FliE^1 inserts between the FliR and FliP^5 subunits, its C-terminal α3 helix makes hydrophobic contacts with α1 of FliR to form the D0-like domain. **(C)** Interaction between FliE^1 and the closed form of FliR (PDB ID: 9K29). The FliR subunit from the 9K29 (plum) was superimposed onto that from 8WKK (purple) structure. In the closed structure of FliR, α3 of FliE collides with helix α1 of FliR as well as the R-loop at the position indicated by the red circle. This collision allows the α1 helix and the R-loop to move outward, thereby dislodging the β-strand (β1) from the β-cap. Consequently, α3 of FliE forms the D0-like domain together with α1 of the open form of FliR.

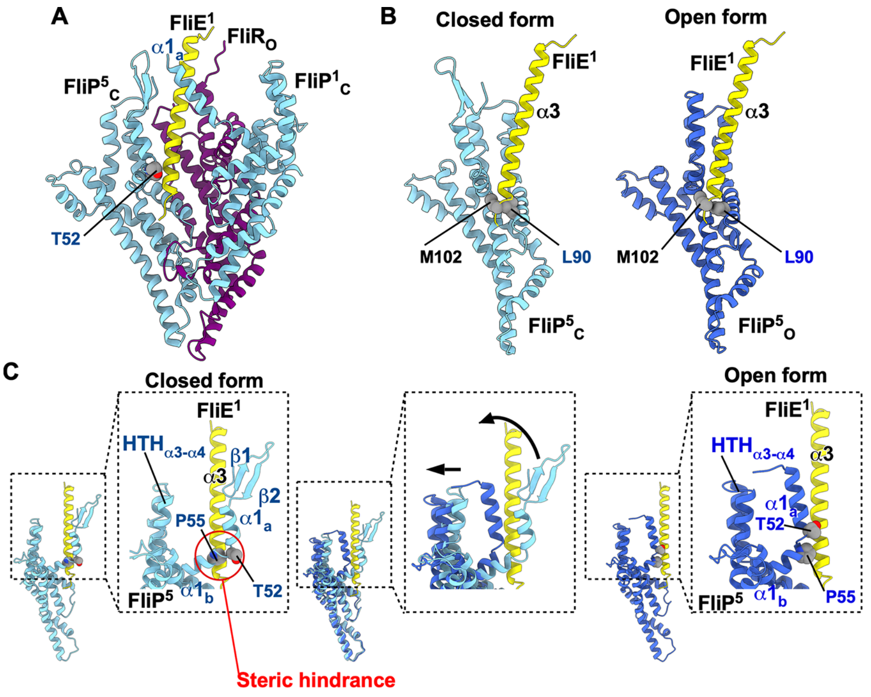

Fig. S15. Interaction between the first FliE subunit and the closed form of the fifth FliP subunit. (A) Superimposition of the equivalent coordinates of the 9K29 (closed form) and 8WKK (open form) structures. Helix $\alpha1_a$ of the closed form of the first FliP subunit ($FliP^1_C$) facilitates the efficient insertion of the first FliE subunit ($FliE^1$) between the open form of FliR ($FliR_O$) and the closed form of the fifth FliP subunit ($FliP^5_C$). **(B)** Interaction between Met-102 of $FliE^1$ and Leu-90 of $FliP^5$. Met-102 of $FliE^1$ makes hydrophobic contact with Leu-90 of $FliP^5$ in both closed and open conformations. This interaction is presumed to reconstitute hydrophobic side-chain interaction networks surrounding the MTSF motif, causing a conformational change of the MTSF motif. **(C)** Interaction between α3 of $FliE^1$ and α1 of $FliP^5$. When α3 of $FliE^1$ is proximal to $\alpha1_a$ of the closed form of $FliP^5$, the C-terminal portion of α3 of $FliE^1$ collides with Thr-52 and Pro-55 of $FliP^5$ at the position indicated by the red circle. The conformational change of the MTSF motif leads to the outward movement of α1 and the helix-turn-helix structure formed by the α3 and α4 helices ($HTH_{\alpha3\text{-}\alpha4}$), resulting in the dislodgment of the β-hairpin of $FliP^5$ from the β-cap. As a result, $FliP^5$ adopts the open conformation and firmly associates with α3 of $FliE^1$.

Table S1. CryoEM data collection, processing, and refinement statistics.

	$FliPQR_{His}$
EMDB	EMD-61993
PDB	9K29
**Data collection and processing**	
Magnification	60,000
Voltage (kV)	300
Electron exposure (e–/Å^2)	80
Defocus range (μm)	-0.7 – -2.2
Pixel size (Å)	0.995
Symmetry imposed	C1
Initial particle images (no.)	164,019
Final particle images (no.)	109,333
Map resolution (Å)	3.01
FSC threshold	0.143
**Refinement**	
Initial model used (PDB code)	7CG4
Model resolution	3.6
FSC threshold	0.143
**Model composition**	
Non-hydrogen atoms	13,230
Protein residues	1722
Ligands	0
***B* factors (Å^2)**	
Protein	100.24
Ligand	0
**R.m.s. deviations**	
Bond length (Å)	0.003
Bond angles (°)	0.706
**Validation**	
MolProbity score	1.54
Clash score	10.54
Rotamer outliers (%)	0
**Ramachandran plot**	
Favored (%)	98.53
Allowed (%)	1.47
Disallowed (%)	0

Table S2. RMSD values of each FliP subunit relative to the first FliP subunit in the 6R69, 9K29, and 8WKK structures (See Fig. 3A).

PDB ID: 6R69 (closed form)		
Reference structure: First FliP subunit		
FliP Subunit	Residues	RMSD (Å)[1]
Second FliP	All	2.041
	Residues 36–64	2.253
Third FliP	All	2.142
	Residues 36–64	2.227
Forth FliP	All	2.418
	Residues 36–64	2.209
Fifth FliP	All	0.812
	Residues 36–64	1.839
**PDB ID: 9K29 (closed from)**		
Reference structure: First FliP subunit		
FliP subunit	Residues	RMSD (Å)
Second FliP	All	2.791
	Residues 36–64	7.079
Third FliP	All	2.704
	Residues 36–64	4.053
Forth FliP	All	2.463
	Residues 36–64	5.923
Fifth FliP	All	2.771
	Residues 36–64	4.077
**PDB ID: 8WKK (closed from)**		
Reference structure: First FliP subunit		
FliP subunit	Residues	RMSD (Å)
Second FliP	All	0.765
	Residues 36–64	0.357
Third FliP	All	0.818
	Residues 36–64	0.447
Forth FliP	All	0.841
	Residues 36–64	0.386
Fifth FliP	All	1.322
	Residues 36–64	0.516

[1] Each root mean square deviation (RMSD) was calculated by aligning each FliP subunit to the first subunit located at the top of the three different cryoEM structures of the FliPQR complex (PDB IDs: 6R69, 9K29, and 8WKK). The RMSD values indicate structural variability among the subunits within each complex, highlighting conformational differences.

Table S3: RMSD values of each subunit of the 9K29 structure in comparison with the corresponding subunit of the 6R69 and 8WKK structures (See Figs. 3B, S7, and S8).

Reference structure: 6R69 (closed form)		
Subunit	Residues	RMSD (Å)
First FliP	All	1.791
	Residues 36–64	3.228
Second FliP	All	1.336
	Residues 36–64	1.011
Third FliP	All	1.365
	Residues 36–64	1.117
Forth FliP	All	1.571
	Residues 36–64	1.904
Fifth FliP	All	1.459
	Residues 36–64	1.945
FliR	All	1.904
	Residues 1–15	1.851
First FliQ	All	0.806
Second FliQ	All	0.797
Third FliQ	All	0.834
Forth FliQ	All	0.908
**Reference structure: 8WKK (open form)**		
Subunit	Residues	RMSD (Å)
First FliP	All	4.608
	Residues 36–64	4.815
Second FliP	All	4.311
	Residues 36–64	7.991
Third FliP	All	4.245
	Residues 36–64	7.625
Forth FliP	All	4.242
	Residues 36–64	6.552
Fifth FliP	All	3.761
	Residues 36–64	5.071
FliR	All	2.193
	Residues 1–15	4.567
First FliQ	All	0.610
Second FliQ	All	0.695
Third FliQ	All	0.711
Forth FliQ	All	0.732

To evaluate structural differences, each subunit of the 9K29 structure was individually aligned with its corresponding subunit in the 6R69 (dark gray) and 8WKK (royal blue) structures. The root mean square deviation (RMSD) was calculated for each pairwise comparison.

Table S4. RMSD values of each FliQ subunit relative to the first FliQ subunit in the 9K29 structure (See Fig. S8A).

**PDB ID: 9K29**		
Reference structure: First FliQ subunit		
Subunit	Residues	RMSD (Å)[1]
Second FliQ	All	0.846
Third FliQ	All	0.879
Forth FliQ	All	1.055

[1] Each root mean square deviation (RMSD) was calculated by aligning each FliQ subunit to the first subunit directly associated with FliR.

Table S5. Calculation of buried surface area (BSA) of Leu-92 in the closed (PDB ID: 9K29) and open (8WKK) conformations of the FliPQR complex using PDBePISA.

		**BSA (Å^2)**					
Interface		FliP^1-FliP^2 (FliP^1)	FliP^2-FliP^3 (FliP^2)	FliP^3-FliP^4 (FliP^3)	FliP^4-FliP^5 (FliP^4)	FliP^5-FliR (FliP^5)	Total
9K29	L92	45.15	46.80	43.08	45.69	54.24	234.96
	A92	36.05	36.76	32.19	27.98	19.62	152.6
	Difference	9.10	10.04	10.89	17.71	34.62	82.36
8WKK	L92	75.95	69.49	58.50	60.49	50.01	314.44
	A92	21.68	24.42	21.10	22.40	20.13	100.73
	Difference	54.27	45.07	37.40	38.09	29.88	204.41

Table S6. Strains and plasmids used in this study.

Strains and Plasmids	Relevant characteristics	Source or reference
Salmonella		
SJW1103	Wild-type for motility and chemotaxis	(9)
SJW1368	Δ(*cheW-flhD*)	(10)
TH10549	Δ*fliP*	K. T. Hughes
**Plasmid**		
pTrc99AFF4	Modified pTrc99A	(11)
pTrcES	Modified pTrc99A	This study
pBAD24E	Modified pBAD24	This study
pKY69	pTrc99AFF4/ His-FliP	(12)
pMKM10001	pTrcES/ FliP + FliQ + FliR-His	This study
pMKM10001(L92A)	pTrcES/ FliP(L92A) + FliQ + FliR-His	This study
pMKM69(ΔMTSF)	pTrc99AFF4/ His-FliP(ΔMTSF)	This study
pMKM69(T62A/S63A)	pTrc99AFF4/ His-FliP(T62A/S63A)	This study
pMKM69(T62G/S63G)	pTrc99AFF4/ His-FliP(T62G/S63G)	This study
pMKM69-G4	pTrc99AFF4/ His-FliP(M61G/T62G/S63G/F64G)	This study
pMKM69-GS2	pTrc99AFF4/ His-FliP(M61G/T62S/S63G/F64S)	This study
pMKM69(W38A)	pTrc99AFF4/ His-FliP(W38A)	This study
pMKM69(W38G)	pTrc99AFF4/ His-FliP(W38G)	This study
pMKM69(L45A)	pTrc99AFF4/ His-FliP(L45A)	This study
pMKM69(F47A)	pTrc99AFF4/ His-FliP(F47A)	This study
pMKM69(L51A)	pTrc99AFF4/ His-FliP(L51A)	This study
pMKM69(T52A)	pTrc99AFF4/ His-FliP(T52A)	This study
pMKM69(F53A)	pTrc99AFF4/ His-FliP(F53A)	This study
pMKM69(L90A)	pTrc99AFF4/ His-FliP(L90A)	This study
pMKM69(L92A)	pTrc99AFF4/ His-FliP(L92A)	This study
pMKM69(L90A/G91A)	pTrc99AFF4/ His-FliP(L90A/G91A)	This study
pMKM69(L90A/L92A)	pTrc99AFF4/ His-FliP(L90A/L92A)	This study
pMKM69(G91A/L92A)	pTrc99AFF4/ His-FliP(G91A/L92A)	This study
pMKM69(L90A/G91A/L92A)	pTrc99AFF4/ His-FliP(L90A/G91A/L92A)	This study
pMKM69(L92A)-SP1	pTrc99AFF4/ His-FliP(P30L/L92A)	This study
pMKM69(L92A)-SP2	pTrc99AFF4/ His-FliP(L45Q/L92A)	This study
pMKM69(L92A)-SP3	pTrc99AFF4/ His-FliP(L92A/R168C)	This study
pMKM69(Δ155-164)	pTrc99AFF4/ His-FliP(Δ155-164)	This study
pMKM69(Δ155-165)	pTrc99AFF4/ His-FliP(Δ155-165)	This study
pMKM69(Δ156-163)	pTrc99AFF4/ His-FliP(Δ156-163)	This study
pMKM69(Δ157-162)	pTrc99AFF4/ His-FliP(Δ157-162)	This study
pMKM701	pBAD24E/ FliR-His	This study
pMKM701(W9A)	pBAD24E/ FliR(W9A)-His	This study
pMKM701(W9G)	pBAD24E/ FliR(W9G)-His	This study

Movie S1. Structural comparison between the closed and open forms of the FliPQR export-channel complex.

Movie S2. FliE assembly mechanism.

SI References

1. D. N. Mastronarde, Automated electron microscope tomography using robust prediction of specimen movements. *J. Struct. Biol.* **152**, 36–51 (2005).
2. K. Yonekura, S. Maki-Yonekura, H. Naitow, T. Hamaguchi, K. Takaba, Machine learning-based real-time object locator/evaluator for cryo-EM data collection. *Commun. Biol.* **4**, 1044 (2021).
3. S. H. W. Scheres, RELION: Implementation of a Bayesian approach to cryo-EM structure determination. *J. Struct. Biol.* **180**, 519–530 (2012).
4. P. Emsley, B. Lohkamp, W. G. Scott, K. Cowtan, Features and development of *Coot*. *Acta Crystallogr. D Struct. Biol.* 66, 486–501 (2010).
5. D. Liebschner *et al*., Macromolecular structure determination using X-rays, neutrons and electrons: recent developments in Phenix. *Acta Crystallogr. D Struct. Biol.* **75**, 861–877 (2019).
6. E. Pettersen *et al*., UCSF ChimeraX: Structure visualizationfor researchers, educators, and developers. *Protein Sci.* **30**, 70–82 (2021).
7. F. Sievers *et al.*, Fast, scalable generation of high-quality protein multiple sequence alignments using Clustal Omega. *Mol. Syst. Biol.* **7**, 539 (2011).
8. H. Ashkenazy, E. Erez, E. Martz, T. Pupko, N. Ben-Tal, ConSurf 2010: calculating evolutionary conservation in sequence and structure of proteins and nucleic acids. *Nucleic Acids Res.* **38**, W529–W533 (2010).
9. S. Yamaguchi *et al*., Genetic analysis of three additional *fla* genes in *Salmonella typhimurium*. *J. Gen. Microbiol.***130**, 3339–3342 (1984).
10. K. Ohnishi, Y. Ohto, S. Aizawa, R. M. Macnab, T. Iino, FlgD is a scaffolding protein needed for flagellar hook assembly in *Salmonella typhimurium*. *J. Bacteriol.* **176**, 2272–2281 (1994).
11. K. Ohnishi, F. Fan, G. J. Schoenhals, M. Kihara, R. M. Macnab, The FliO, FliP, FliQ, and FliR proteins of *Salmonella typhimurium*: putative components for flagellar assembly. *J. Bacteriol.* **179,** 6092–6099 (1997).
12. T. Fukumura *et al*., Assembly and stoichiometry of the core structure of the bacterial flagellar type III export gate complex. *PLoS Biol*. **15**, e2002281 (2017).
